# Treatment and outcomes in children with multidrug-resistant tuberculosis: A systematic review and individual patient data meta-analysis

**DOI:** 10.1371/journal.pmed.1002591

**Published:** 2018-07-11

**Authors:** Elizabeth P. Harausz, Anthony J. Garcia-Prats, Stephanie Law, H. Simon Schaaf, Tamara Kredo, James A. Seddon, Dick Menzies, Anna Turkova, Jay Achar, Farhana Amanullah, Pennan Barry, Mercedes Becerra, Edward D. Chan, Pei Chun Chan, Domnica Ioana Chiotan, Aldo Crossa, Peter C. Drobac, Lee Fairlie, Dennis Falzon, Jennifer Flood, Medea Gegia, Robert M. Hicks, Petros Isaakidis, SM Kadri, Beate Kampmann, Shabir A. Madhi, Else Marais, Andrei Mariandyshev, Ana Méndez-Echevarría, Brittany Kathryn Moore, Parpieva Nargiza, Iveta Ozere, Nesri Padayatchi, Saleem- ur-Rehman, Natasha Rybak, Begoña Santiago-Garcia, N. Sarita Shah, Sangeeta Sharma, Tae Sun Shim, Alena Skrahina, Antoni Soriano-Arandes, Martin van den Boom, Marieke J. van der Werf, Tjip S. van der Werf, Bhanu Williams, Elena Yablokova, Jae-Joon Yim, Jennifer Furin, Anneke C. Hesseling, for the Collaborative Group for Meta-Analysis of Paediatric Individual Patient Data in MDR-TB

**Affiliations:** 1 Desmond Tutu TB Centre, Department of Paediatrics and Child Health, Faculty of Medicine and Health Sciences, Stellenbosch University, Tygerberg, South Africa; 2 Military HIV Research Program, Bethesda, Maryland, United States of America; 3 Montreal Chest Institute, McGill University, Montreal, Quebec, Canada; 4 Cochrane South Africa, South African Medical Research Council, Cape Town, South Africa; 5 Centre for International Child Health, Imperial College, London, United Kingdom; 6 Imperial College Healthcare NHS Trust, Institute of Clinical Trials and Methodology, London, United Kingdom; 7 Manson Unit, Médecins Sans Frontières (MSF), London, United Kingdom; 8 The Indus Hospital, Karachi, Pakistan; 9 California Department of Public Health, Sacramento, California, United States of America; 10 Partners In Health, Harvard Medical School, and Brigham and Women's Hospital, Boston, Massachusetts, United States of America; 11 Denver Veterans Affairs Medical Center, National Jewish Health, Denver, Colorado, United States of America; 12 Division of Chronic Infectious Disease, Centers for Disease Control, Taipei, Taiwan; 13 Epidemiological Surveillance Department, Romanian National TB Program, Bucharest, Romania; 14 New York City Department of Health and Mental Hygiene, New York, New York, United States of America; 15 Wits Reproductive Health & HIV Institute (WRHI), University of the Witwatersrand, Johannesburg, South Africa; 16 Laboratories, Diagnostics and Drug Resistance Unit, Global TB Programme, World Health Organization, Geneva, Switzerland; 17 Technical Support Coordination, Global TB Programme, World Health Organization, Geneva, Switzerland; 18 Albert Einstein College of Medicine, Bronx, New York, United States of America; 19 Médecins Sans Frontières (MSF)/Doctors Without Borders, Mumbai, India; 20 Disease Control, Directorate of Health Services, Kashmir, India; 21 Paediatric Infection & Immunity, Centre of International Child Health, Imperial College London, London, United Kingdom; 22 Vaccines & Immunity Theme, MRC Unit The Gambia, Banjul, The Gambia; 23 Medical Research Council: Respiratory and Meningeal Pathogens Research Unit, and Department of Science and Technology/National Research Foundation: Vaccine Preventable Diseases, University of the Witwatersrand, Johannesburg, South Africa; 24 Department of Clinical Microbiology and Infectious Diseases, University of the Witwatersrand and the National Health Laboratory Services, Johannesburg, South Africa; 25 Northern State Medical University, Arkhangelsk, Russian Federation; 26 Pediatric, Infectious and Tropical Diseases Department, Hospital La Paz, Madrid, Spain; 27 Division of Global HIV and TB, U.S. Centers for Disease Control and Prevention, Atlanta, Georgia, United States of America; 28 Republican Scientific Medical Center of Phtiziology and Pulmonology, Ministry of Health, Tashkent, Uzbekistan; 29 Riga Eastern Clinical University Hospital, Centre for Tuberculosis and Lung Diseases, Riga, Latvia; 30 CAPRISA, MRC TB-HIV Pathogenesis Unit, Durban, South Africa; 31 Director Health Services, Kashmir, India; 32 Alpert Medical School of Brown University, Providence, Rhode Island, United States of America; 33 Pediatric Infectious Diseases Unit, Instituto de Investigación Sanitaria Gregorio Marañón, Hospital General Universitario Gregorio Marañón, Madrid, Spain; 34 Department of Pediatrics, National Institute of Tuberculosis and Respiratory Diseases, New Delhi, India; 35 Department of Pulmonary and Critical Care Medicine, University of Ulsan College of Medicine, Asan Medical Center, Seoul, South Korea; 36 The Republican Research and Practical Centre for Pulmonology and TB, Minsk, Belarus; 37 Pediatric Infectious Diseases and Immunodeficiencies Unit, Unit of International Health-Tuberculosis Drassanes-Vall Hebron, Hospital Universitari Vall d'Hebron, Barcelona, Spain; 38 Joint Tuberculosis, HIV & Viral Hepatitis Programme, WHO Regional Office for Europe, Copenhagen, Denmark; 39 Disease Programme Tuberculosis, European Centre for Disease Prevention and Control, Stockholm, Sweden; 40 University Medical Center Groningen, Groningen, the Netherlands; 41 Northwick Park Hospital, London Northwest Healthcare NHS Trust, London, United Kingdom; 42 Division of Pulmonary and Critical Care Medicine, Department of Internal Medicine, Seoul National University College of Medicine, Seoul, South Korea; 43 Department of Global Health and Social Medicine, Harvard Medical School, Boston, Massachusetts, United States of America; University of California, San Francisco, UNITED STATES

## Abstract

**Background:**

An estimated 32,000 children develop multidrug-resistant tuberculosis (MDR-TB; *Mycobacterium tuberculosis* resistant to isoniazid and rifampin) each year. Little is known about the optimal treatment for these children.

**Methods and findings:**

To inform the pediatric aspects of the revised World Health Organization (WHO) MDR-TB treatment guidelines, we performed a systematic review and individual patient data (IPD) meta-analysis, describing treatment outcomes in children treated for MDR-TB. To identify eligible reports we searched PubMed, LILACS, Embase, The Cochrane Library, PsychINFO, and BioMedCentral databases through 1 October 2014. To identify unpublished data, we reviewed conference abstracts, contacted experts in the field, and requested data through other routes, including at national and international conferences and through organizations working in pediatric MDR-TB. A cohort was eligible for inclusion if it included a minimum of three children (aged <15 years) who were treated for bacteriologically confirmed or clinically diagnosed MDR-TB, and if treatment outcomes were reported. The search yielded 2,772 reports; after review, 33 studies were eligible for inclusion, with IPD provided for 28 of these. All data were from published or unpublished observational cohorts. We analyzed demographic, clinical, and treatment factors as predictors of treatment outcome. In order to obtain adjusted estimates, we used a random-effects multivariable logistic regression (random intercept and random slope, unless specified otherwise) adjusted for the following covariates: age, sex, HIV infection, malnutrition, severe extrapulmonary disease, or the presence of severe disease on chest radiograph. We analyzed data from 975 children from 18 countries; 731 (75%) had bacteriologically confirmed and 244 (25%) had clinically diagnosed MDR-TB. The median age was 7.1 years. Of 910 (93%) children with documented HIV status, 359 (39%) were infected with HIV. When compared to clinically diagnosed patients, children with confirmed MDR-TB were more likely to be older, to be infected with HIV, to be malnourished, and to have severe tuberculosis (TB) on chest radiograph (*p* < 0.001 for all characteristics). Overall, 764 of 975 (78%) had a successful treatment outcome at the conclusion of therapy: 548/731 (75%) of confirmed and 216/244 (89%) of clinically diagnosed children (absolute difference 14%, 95% confidence interval [CI] 8%–19%, *p* < 0.001). Treatment was successful in only 56% of children with bacteriologically confirmed TB who were infected with HIV who did not receive any antiretroviral treatment (ART) during MDR-TB therapy, compared to 82% in children infected with HIV who received ART during MDR-TB therapy (absolute difference 26%, 95% CI 5%–48%, *p* = 0.006). In children with confirmed MDR-TB, the use of second-line injectable agents and high-dose isoniazid (15–20 mg/kg/day) were associated with treatment success (adjusted odds ratio [aOR] 2.9, 95% CI 1.0–8.3, *p* = 0.041 and aOR 5.9, 95% CI 1.7–20.5, *p* = 0.007, respectively). These findings for high-dose isoniazid may have been affected by site effect, as the majority of patients came from Cape Town. Limitations of this study include the difficulty of estimating the treatment effects of individual drugs within multidrug regimens, only observational cohort studies were available for inclusion, and treatment decisions were based on the clinician’s perception of illness, with resulting potential for bias.

**Conclusions:**

This study suggests that children respond favorably to MDR-TB treatment. The low success rate in children infected with HIV who did not receive ART during their MDR-TB treatment highlights the need for ART in these children. Our findings of individual drug effects on treatment outcome should be further evaluated.

## Abstract 2

In a systematic review and meta-analysis conducted to inform World Health Organization guidelines, Elizabeth Harausz and colleagues investigate different treatment regimens and outcomes for children with multidrug-resistant tuberculosis.

## Author summary

**Why was this study done?:**

- Treatment for multidrug-resistant tuberculosis (MDR TB) affects 32,000 children per year, requires longer treatment with much more toxic medications than drug-susceptible tuberculosis. Unfortunately, little is know about the optimal treatment for children with MDR TB.
- This study reviewed treatment and outcome data from children around the world in order to better understand the management of MDR-TB in children.
- This study also sought to understand the risk factors for poor treatment outcomes in children with MDR-TB.
- This study informed the World Health Organization guidelines on treatment of MDR-TB in children.

**What did the researchers do and find?:**

- We performed a systematic review and individual patient data meta-analysis on clinical characteristics and treatment outcomes on 975 children from across 18 countries.
- Children were analyzed in two separate groups, those with bacteriologically confirmed MDR-TB and those who were clinically diagnosed with MDR-TB.
- We found that, in general, children do well when treated with the second-line MDR-TB medications (78% overall had successful treatment outcomes), despite the fact that there was a high burden of severe disease.
- Malnutrition and not being treated for HIV (if the child was HIV-positive) during TB treatment significantly increased the risk of poor outcomes.
- Second-line injectable agents and high-dose isoniazid were associated with treatment success. However, a high proportion of children with non-severe disease who received no second-line injectable agents still did well; therefore, children with non-severe disease may be able to be spared from these toxic medications.

**What do these findings mean?:**

- Consideration should be given to using high-dose isoniazid in treatment regimens, and if children have non-severe disease, the possibility of excluding second-line injectable agents from the treatment regimen should be considered.
- HIV treatment should be started as soon as is possible, and malnutrition should be aggressively treated.

## Introduction

Almost 500,000 people developed multidrug-resistant tuberculosis (MDR-TB) (defined as *Mycobacterium tuberculosis* with resistance to at least isoniazid and rifampin) in 2015 [1]. Despite the fact that as many as 32,000 children younger than 15 years of age develop MDR-TB globally each year, little is known about the optimal treatment for children with MDR-TB [2]. The diagnosis and treatment of MDR-TB in children is challenging: it can be difficult to bacteriologically confirm the diagnosis because of difficulties in collecting respiratory samples in younger children and in children who frequently have paucibacillary (smear- or culture-negative) disease [3].

Treatment of MDR-TB is difficult as well, requiring the use of second-line medications in regimens much longer (often lasting 18–20 months) than those for drug-susceptible disease [4]. These regimens are frequently hard to tolerate, particularly in children, due to the length of treatment, drug toxicity, and the lack of child-friendly formulations. However, unlike adults, children have a wide spectrum of tuberculosis (TB) disease, from limited disease with low bacillary burden, to more severe adult-type disease (e.g., cavitating pulmonary TB) with a higher bacillary load [3]. Children with less severe disease may not require a treatment regimen as intensive as children with more severe disease or adults. Therefore, they may be able to be spared from these long, toxic regimens [5].

A previous systematic review of 315 children from eight cohorts from five countries reported successful treatment outcomes in 82% of children with clinically or bacteriologically diagnosed MDR-TB [6]. Clinical or treatment factors associated with outcomes in children with MDR-TB have been evaluated in a few individual studies but have not been well characterized in large, geographically diverse cohorts. The small size of individual published cohorts limits the precision in assessing the impact of clinical factors on outcomes, and their lack of geographic diversity restricts the generalizability of findings. Study-level meta-analyses limit the useful inferences that may be drawn on the associations between outcomes and the treatment, and limit the adjustment for confounding or interaction that would be possible from the analysis of individual patient data (IPD). A more rigorous evidence base is needed to help guide the management of MDR-TB treatment in children globally. Given the paucibacillary nature of TB in most children and the potential for certain children to receive less intensive and less toxic treatment regimens, an understanding of risk factors for poor outcomes across settings is important for designing future treatment regimens.

In order to provide this stronger evidence base and to inform the revised 2016 World Health Organization (WHO) MDR-TB treatment guidelines, we undertook a systematic review and IPD meta-analysis to describe treatment outcomes among children with confirmed or clinically diagnosed MDR-TB, and to characterize demographic, clinical, and treatment factors associated with treatment outcomes [4].

## Methods

### Eligibility criteria

A cohort was eligible for inclusion in this IPD meta-analysis if it included a minimum of three children (aged <15 years) within a defined treatment cohort who were treated for bacteriologically confirmed (“confirmed”) or clinically diagnosed pulmonary or extrapulmonary MDR-TB, and for whom treatment outcomes were reported, using standard 2014 WHO MDR-TB outcome definitions (Table 1) [7,8]. All cohorts containing children included in a previous, largely adult systematic review and IPD meta-analysis of MDR-TB were also considered eligible; those data were accepted as one data set, even if that data set contained fewer than three children from a defined geographic area [9]. In order to be defined as having “confirmed” MDR-TB, there needed to be bacteriological confirmation with documented resistance to both isoniazid and rifampicin on genotypic or phenotypic testing. The basis for a clinical diagnosis of TB is described in Table 1. Eligibility criteria were applied at the individual level, so that studies reporting on both adults and children could be considered eligible if they otherwise met the specified criteria. Both published and unpublished data were included, without date restrictions. Eligible study designs included controlled and noncontrolled retrospective and prospective studies and case series. Reports written in English, Spanish, French, Dutch, and Russian were included. Studies that used only combinations of rifampin, isoniazid, pyrazinamide, ethambutol, or streptomycin to treat MDR-TB were excluded, as this is considered inadequate therapy.

**Table 1:** Tuberculosis case definitions and treatment outcome definitions.

TB case definitions	
Bacteriologically confirmed TB	A case of TB in a patient from whom a biological specimen was positive by smear microscopy^1^, culture, or WHO-approved rapid diagnostics (including GeneXpert MTB/RIF).
Clinically diagnosed TB	A case of TB in a patient who does not fulfill the criteria for bacteriological confirmation but who has been diagnosed with TB disease by a clinician or other medical practitioner who has decided to give the patient a full course of TB treatment. This definition includes cases diagnosed on the basis of radiographic abnormalities or suggestive histology and extrapulmonary cases without laboratory confirmation.
TB source case	A case of infectious TB (usually sputum smear- or culture-positive) in a person who transmits infection to one or more other individuals.
MDR-TB outcome definitions	
Cured	Treatment completed as recommended by the national policy without evidence of failure, and three or more consecutive cultures taken at least 30 days apart are negative after the intensive phase of treatment.
Treatment completed	Treatment completed as recommended by the national policy without evidence of failure, but no record that three or more consecutive cultures taken at least 30 days apart are negative after the intensive phase of treatment.
Treatment failed	Treatment terminated or need for permanent regimen change of at least two anti-TB drugs because of • lack of conversion by the end of the intensive phase, • bacteriological reversion in the continuation phase after conversion to negative, • evidence of additional acquired resistance to fluoroquinolones or second-line injectable drugs, or • ADRs.
Died	A patient who dies for any reason during the course of treatment.
Lost to follow-up	A patient whose treatment was interrupted for two consecutive months or more.
Not evaluated	A case of TB in a patient for whom no treatment outcome is assigned. This includes patients “transferred out” to another treatment unit and whose treatment outcome is unknown.
Treatment success	The sum of cured and treatment completed.

Adapted from: Guidance for national tuberculosis programmes on the management of tuberculosis in children: second edition. WHO, 2014 [7].

^1^For this study, participants had to have MDR-TB confirmed by culture or WHO-approved rapid diagnostics; smear microscopy was not sufficient to be classified as bacteriologically confirmed.

Abbreviations: ADR, adverse drug reaction; MDR-TB, multidrug-resistant tuberculosis; MTB/RIF, *Mycobacterium tuberculosis*/rifampin; TB, tuberculosis; WHO, World Health Organization.

### Identifying primary reports

In order to identify eligible reports, we searched PubMed, LILACS (Latin American and Caribbean Health Sciences Literature), Embase, The Cochrane Library, PsychINFO, and BioMedCentral databases through 1 October 2014, with a search strategy using a combination of the search terms “tuberculosis,” “multidrug resistance,” “MDR-TB,” “multidrug-resistant,” and “children,” both as exploded MeSH (Medical Subject Headings) headings and free-text terms, and without language restriction. The specific search strategies for Pubmed and Embase are presented in S1 Table. We also reviewed conference abstracts from the annual World Lung Health Conferences of the International Union Against Tuberculosis and Lung Diseases (The Union).

To identify additional published and unpublished data, we contacted experts in the field of pediatric MDR-TB. We also requested data through other routes, including at national and international conferences and training events, and through international and in-country organizations working in pediatric MDR-TB, including the Sentinel Project on Pediatric Drug-Resistant Tuberculosis, the WHO Childhood TB Sub-Group, Médecins Sans Frontières (MSF), the United States Centers for Disease Control and Prevention (CDC) and European Centre for Disease Prevention and Control (ECDC), The Union, and the National Institutes of Health (NIH).

### Report selection and review

All abstracts were screened by EPH and a researcher with Cochrane South Africa to select full text reports to review. All full text reports were reviewed independently by two reviewers (EPH, AJGP, HSS, JF, ACH) to assess for eligibility, except reports in Russian, French, Dutch, and Spanish, which were reviewed by a single reviewer (AT, EPH, ACH, and JF, respectively). Disagreements about study selection were resolved by a third reviewer. If eligibility was unclear because of missing information, two attempts were made to contact authors of the primary report. After two unsuccessful attempts, reports were excluded.

The quality of individual studies was described using a modified version of the Newcastle-Ottawa approach for cohort studies adapted for use in pediatric MDR-TB [10]. An example of our grading approach is provided in S2 Table, followed by the grading of the individual studies.

### Data sharing and abstraction

The authors of all eligible studies and cohorts were contacted to request IPD. De-identified IPD were included following written agreement from the original authors, or the lead clinical physician in the case of unpublished data.

Data were requested on factors that could influence treatment decision and outcome, including:

Participants: age, sex, nutritional status, HIV status and antiretroviral treatment (ART), adult MDR-TB source case information, bacteriologically confirmed versus clinical diagnosis (Table 1), disease site (pulmonary or extrapulmonary), severity of disease on chest radiograph, drug susceptibility test (DST) results, and history of previous TB episodes.

Intervention: use of individual drugs and the duration of drug use in the treatment regimen.

Outcomes: acid-fast bacilli (AFB) smear microscopy (smear) and culture conversion, adverse events, and WHO-defined treatment outcomes, including cure, treatment completion, treatment failure, loss to follow-up, not evaluated, and death (see Table 1 for definitions) [7].

Severity of TB disease on the chest radiograph was graded independently by two reviewers (EPH, ACH) as either severe or non-severe, based on the reported chest radiographic findings using adapted Wiseman criteria [11]; disagreements were arbitrated by a third reviewer (HSS). Malnutrition was defined as being severely underweight for age (weight-for-age-adjusted z-score of less than −3) or malnourished, as per attending clinician’s clinical assessment, including the presence of nutritional edema. Severe extrapulmonary TB was defined as TB meningitis, miliary TB, abdominal, osteoarticular, or “disseminated TB disease” (this diagnosis was given in 11 children, without further details). The primary authors of all included reports were contacted to resolve any queries.

Although we collected information on adverse effects, we were unable to complete formal analyses given the limitations in the data.

### Statistical analysis

Data from children with confirmed pre-extensively drug-resistant (pre-XDR)-TB (MDR-TB with additional resistance to either a fluoroquinolone or a second-line injectable agent) were combined with those from children with MDR-TB. Children with confirmed extensively drug-resistant TB (XDR-TB, defined as MDR-TB with additional resistance to both a fluoroquinolone and a second-line injectable agent) were excluded from analysis and will be reported on separately. This was done because patients with XDR-TB generally have very different treatment requirements, such that their outcomes would not be representative of the MDR-TB and pre-XDR-TB cohorts. When analyzing the association between individual drug use and treatment success, treatment outcomes were dichotomized as either successful or unsuccessful and stratified by confirmed versus clinically diagnosed MDR-TB. Successful treatment outcome was defined as cure or treatment completion (Table 1). Unsuccessful outcome was defined as treatment failure or death. Children who were lost to follow-up or not evaluated were excluded from analyses.

With respect to the analysis of the estimates of the association between individual antituberculosis medications (used as part of a multidrug regimen) and treatment success, in order to obtain adjusted estimates, we used a random-effects multivariable logistic regression (random intercept and random slope, unless specified otherwise) with adaptive quadrature approximation. Our models for predictors of drug effect on TB treatment outcome only included those children with bacteriologically confirmed MDR-TB. Patients were considered to be clustered within cohorts such that intercepts and slopes of the main exposure variables were allowed to vary across cohorts. This was done to account for unmeasured differences in patient populations and other site-specific characteristics. Estimates were adjusted for the following covariates: age (as a continuous variable), sex, HIV infection, malnutrition, severe extrapulmonary disease, or the presence of severe disease on chest radiograph. There were nine sites that did not report any information on patients' nutritional status (*n* = 44 children) and one site that did not report on the severity of extrapulmonary disease (*n* = 2 children). For the patients at sites that had no data from which to estimate a mean value, their missing values were replaced in the analysis by the mean value of the variable in the entire sample. At sites where most patients had these values reported, for individual patients who had missing data on HIV status (*n* = 65), sex (*n* = 3), severe extrapulmonary disease (*n* = 68), and malnutrition (*n* = 43), missing values were replaced by their respective site's mean value of the variable. Children from countries with very low HIV prevalence who did not have an HIV test were assumed to be HIV uninfected following consultation with site investigators. A *p*-value of 0.05 was taken as the limit of statistical significance. All statistical analyses were performed using SAS software (version 9.3, SAS Institute, Cary, NC).

### Ethical considerations

A protocol prespecified the study rationale and methods (S2 Text). Ethics approval was provided by the Health Research Ethics Committee of the Faculty of Medicine and Health Sciences, Stellenbosch University (reference number X14/09/020). The collection of the original data and sharing of those data was approved by the appropriate oversight body at each contributor’s local institution, including the use of unpublished data, prior to release of data to the team.

For IPD contributed by the US CDC, Atlanta, GA, Institutional Review Board approval was obtained from the South African Medical Research Council Ethics Committee and the Human Research Ethics Committee of University of the Witwatersrand in Johannesburg and was determined to be routine disease surveillance by the US CDC.

## Results

### Search results and report selection

**Fig 1:**
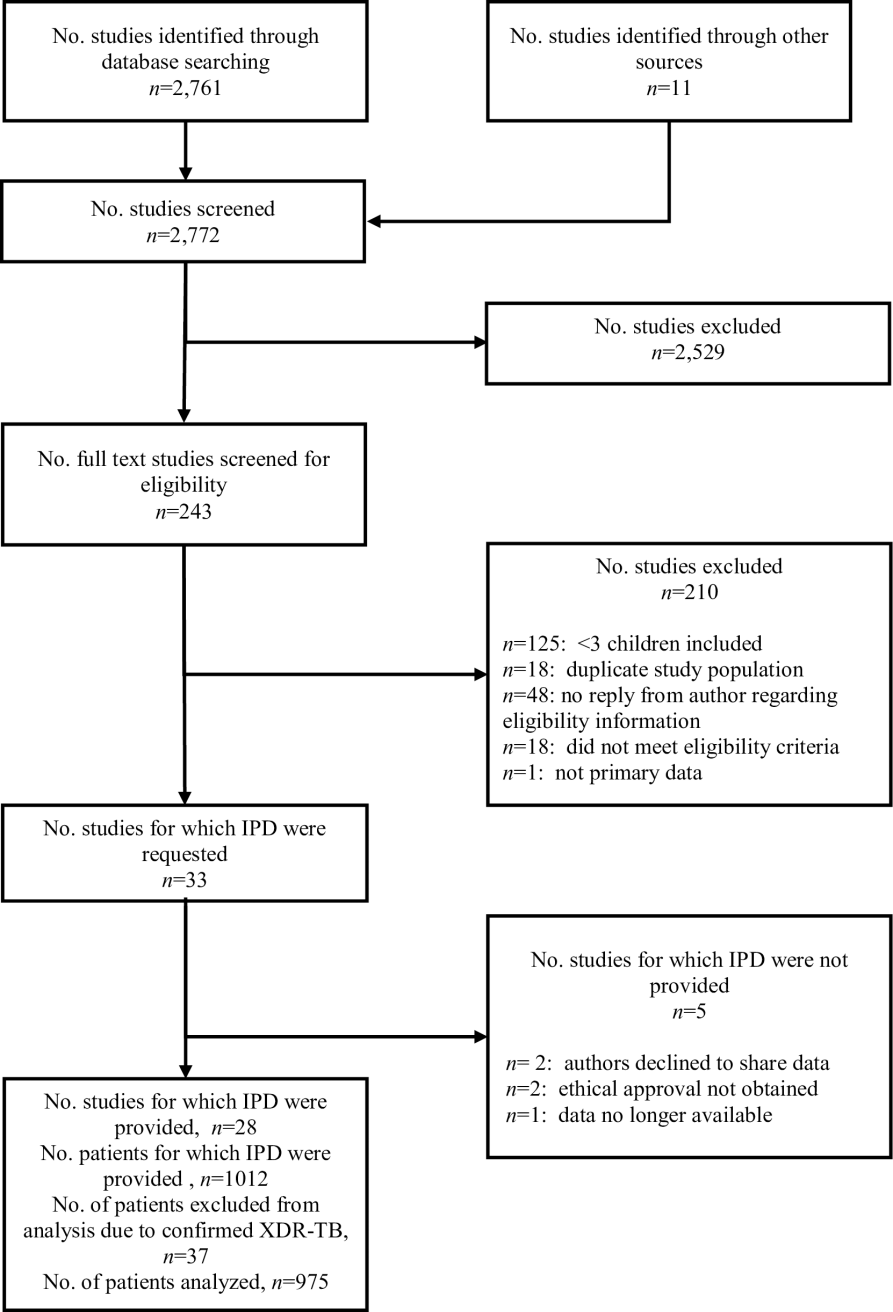
Study selection. IPD, individual patient data; XDR-TB, extensively drug resistant tuberculosis.

The search results and report selection are summarized in Fig 1 (see S1 Text for PRISMA checklist). The search yielded 2,772 reports; after screening of abstracts and review of the full texts, 33 studies were eligible for inclusion, with IPD provided for 28 of these [9,12–33] (Fig 1). Overall, the studies were noted to be of low quality, given the lack of any randomized controlled trials (see S3 Table for overview of studies). Although we are unable to quantify the pediatric data that were not included, we believe they were minimal. The largest group of studies from which data were excluded were the studies in which there was no reply from the authors, so it is unknown exactly how many children may have been excluded. However, these were almost all studies with mainly adult patients, but inclusion of children could not be ruled out with certainty based on reading these articles.

### Report characteristics

**Fig 2:**
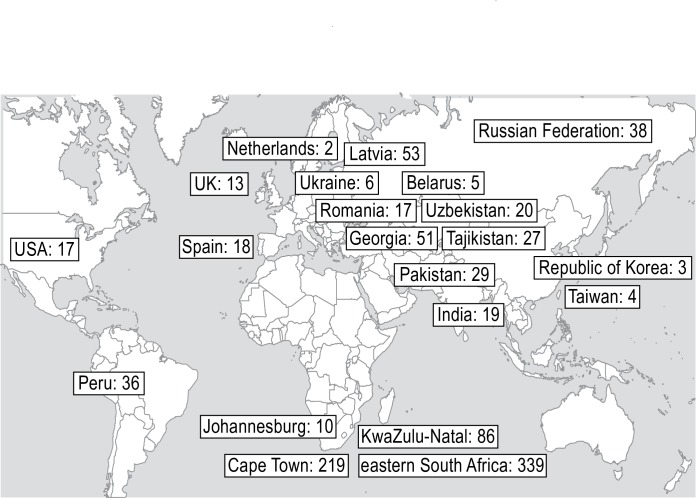
Geographic distribution of patients. Locations of patients included in the individual patient data meta-analysis. The number indicates the number of participants included from each location.

Data sets were received from sites in 18 countries with a broad geographic distribution (Fig 2), with the majority from Africa. Nine countries (Belarus, India, Pakistan, Peru, Russian Federation, South Africa, Tajikistan, Ukraine, Uzbekistan) were among the 30 high-burden MDR-TB countries [1]. Details of published and unpublished cohorts included are presented in S3 Table. Eight percent of children were treated between 1990 and 2004, 67% of children were treated between 2005 and 2009, and 25% were treated from 2010 and later.

### Summary patient data and outcomes

The 28 cohorts included data from 1,012 patients. All data were from observational studies in which children were treated with the local standard of care. Thirty-seven children with confirmed XDR-TB were excluded from analyses; therefore, data from 975 children were analyzed. Of the 975 children, 731 (75%) had confirmed MDR-TB by DST and 244 (25%) had clinically diagnosed MDR-TB. Most data predated the rollout of GeneXpert MTB/RIF; the vast majority of confirmed patients were diagnosed using culture. Of the 731 children with a confirmed diagnosis, 68 (9.3%) had pre-XDR-TB (36 with MDR-TB with additional fluoroquinolone resistance, and 32 with additional second-line injectable resistance). It should be noted that 68% of confirmed patients had no additional information regarding resistance to fluoroquinolones and/or second-line injectable agents. Of the 244 children with a clinical diagnosis of MDR-TB, 164 (67%) had clinical TB with a known close source case with DST-confirmed MDR-TB, 14 (6%) had failure of first-line therapy (first-line oral anti-TB drugs are isoniazid, rifampin, ethambutol, pyrazinamide, rifabutin, and rifapentine [34]) despite documented good adherence, and one child had a clinical diagnosis of TB with a known MDR-TB source case, in addition to treatment failure on first-line TB drugs despite good adherence. The basis for the clinical diagnosis of MDR-TB was not specified in 65 patients (27%).

Key demographic and clinical characteristics, stratified by confirmed versus clinically diagnosed MDR-TB, are shown in Table 2. The median age was 7.1 years (IQR 2.6–11.7); 429 (44%) were males. HIV status was documented in 910 (93%) children, of whom 359 (39%) were infected with HIV. Children with confirmed MDR-TB were more likely to be older, to be infected with HIV, to be malnourished, and to have severe TB on chest radiograph.

**Table 2:** Demographic and clinical characteristics among children with MDR-TB.

Characteristic	All children *n* = 975 (%)	Bacteriologically confirmed MDR or Pre-XDR-TB *n* = 731 (%)	Clinically diagnosed MDR-TB *n* = 244 (%)	*p*-value^d^
Age (years)				
<5	399 (41)	240 (33)	159 (65)	<0.001
5 to <10	234 (24)	178 (24)	56 (23)	
10 to <15	342 (35)	313 (43)	29 (12)	
Median age	7.1, IQR 2.6–11.7	8.5, IQR 3.4–12.2	3.6, IQR 1.9–7.0	
Sex				
Female	543 (56)	414 (57)	129 (53)	0.570
Male	429 (44)	315 (43)	114 (47)	
Unknown	3 (0.3)	2 (0.3)	1 (0.4)	
HIV status				
Infected with HIV	359 (37)	323 (44)	36 (15)	<0.001
Not infected with HIV	551 (57)	356 (49)	195 (80)	
Unknown	65 (7)	52 (7)	13 (5)	
Malnourished^a^				
Yes	332 (34)	276 (38)	56 (23)	<0.001
No	556 (57)	381 (52)	175 (72)	
Unknown	87 (9)	74 (10)	13 (5)	
Severe disease on chest radiograph				
Yes	474 (49)	407 (56)	67 (28)	<0.001
No	294 (30)	168 (23)	126 (52)	
Unknown	207 (21)	156 (21)	51 (21)	
Severe extrapulmonary disease^b^	127 (13)	103 (14)	24 (10)	0.031
Site of disease				
Pulmonary only	710 (73)	526 (72)	184 (75)	0.002
Extrapulmonary only	99 (10)	67 (9)	32 (13)	
Both extrapulmonary and pulmonary	152 (16)	130 (18)	22 (9)	
Unknown	14 (1)	8 (1)	6 (3)	
Extrapulmonary disease sites^c^				
Meningitis	34 (14)	23 (12)	11 (20)	
Miliary	34 (12)	29 (15)	5 (9)	
Bone/joint (including spine)	25 (10)	20 (10)	5 (9)	
Pleural	19 (8)	15 (8)	4 (7)	
Urogenital	1 (0.4)	1 (0.5)	0	
Abdominal	53 (21)	48 (24)	5 (9)	
Skin	1 (0.4)	1 (0.5)	0	
Disseminated disease not otherwise specified	11 (4)	10 (5)	1 (2)	

^a^Malnourished was defined as being underweight or malnourished by clinical diagnosis, having nutritional edema, or having low weight for age (weight-for-age-adjusted z-score of less than **−**3).

^b^Severe extrapulmonary disease was defined as meningitis, miliary, abdominal, osteoarticlar, and disseminated disease not otherwise specified.

^c^Disease sites are not mutually exclusive; one child could have multiple disease sites. Denominator is children with only extrapulmonary and with both pulmonary and extrapulmonary disease sites.

^d^*p*-value represents differences in characteristics between clinically diagnosed and bacteriologically confirmed cohorts.

Abbreviations: MDR-TB, multidrug-resistant tuberculosis, pre-XDR-TB, pre-extensively drug-resistant tuberculosis; XDR-TB, extensively drug resistant tuberculosis.

**Fig 3:**
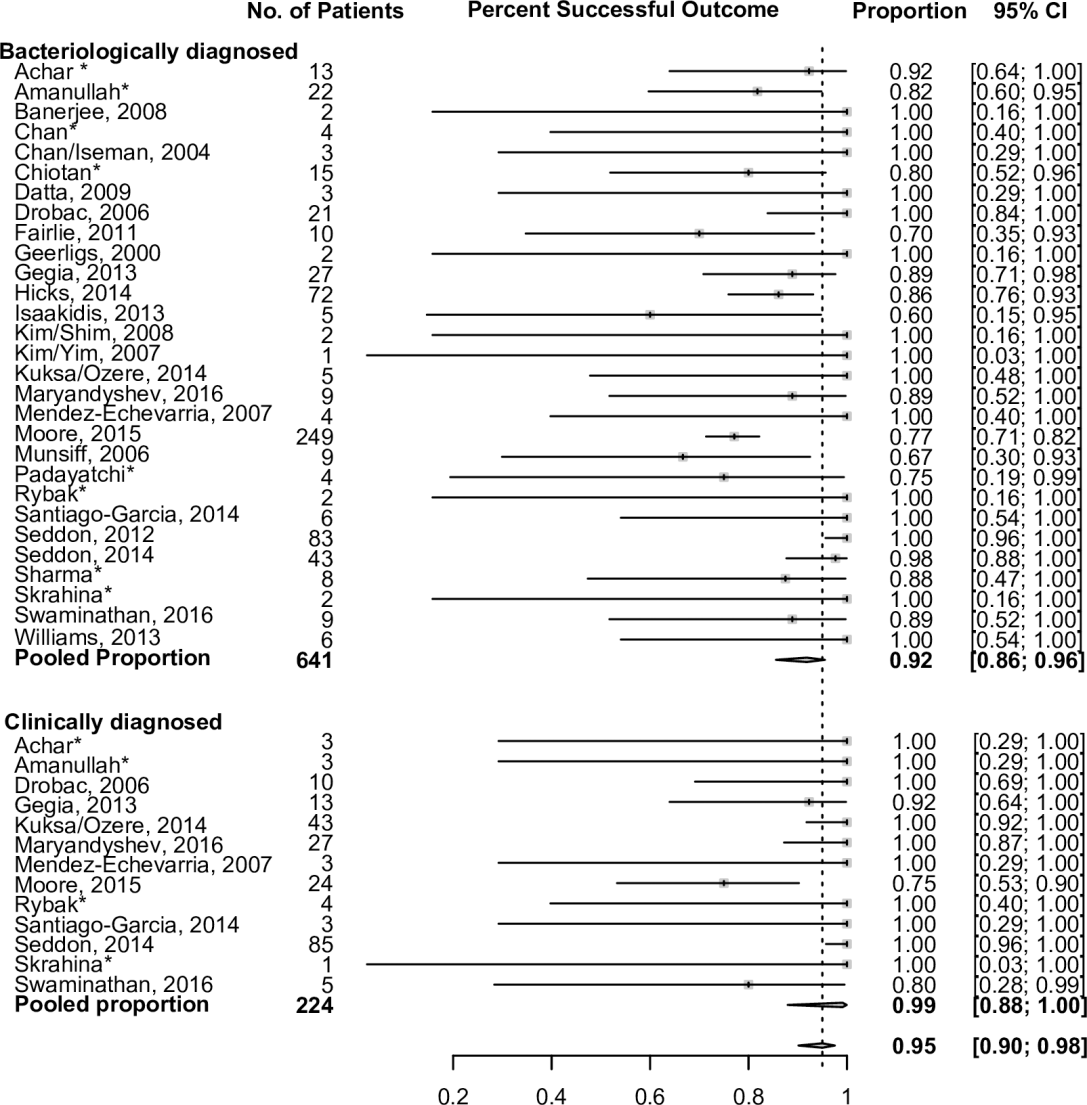
Proportion of patients achieving successful treatment outcomes, stratified by method of diagnosis, by site. Excludes lost to follow-up and cases that did not have an outcome. Results estimated via random effects modeling to account for clustering by cohort. The 95% confidence limits were estimated using exact (Clopper-Pearson) method. *Unpublished data.

**Fig 4:**
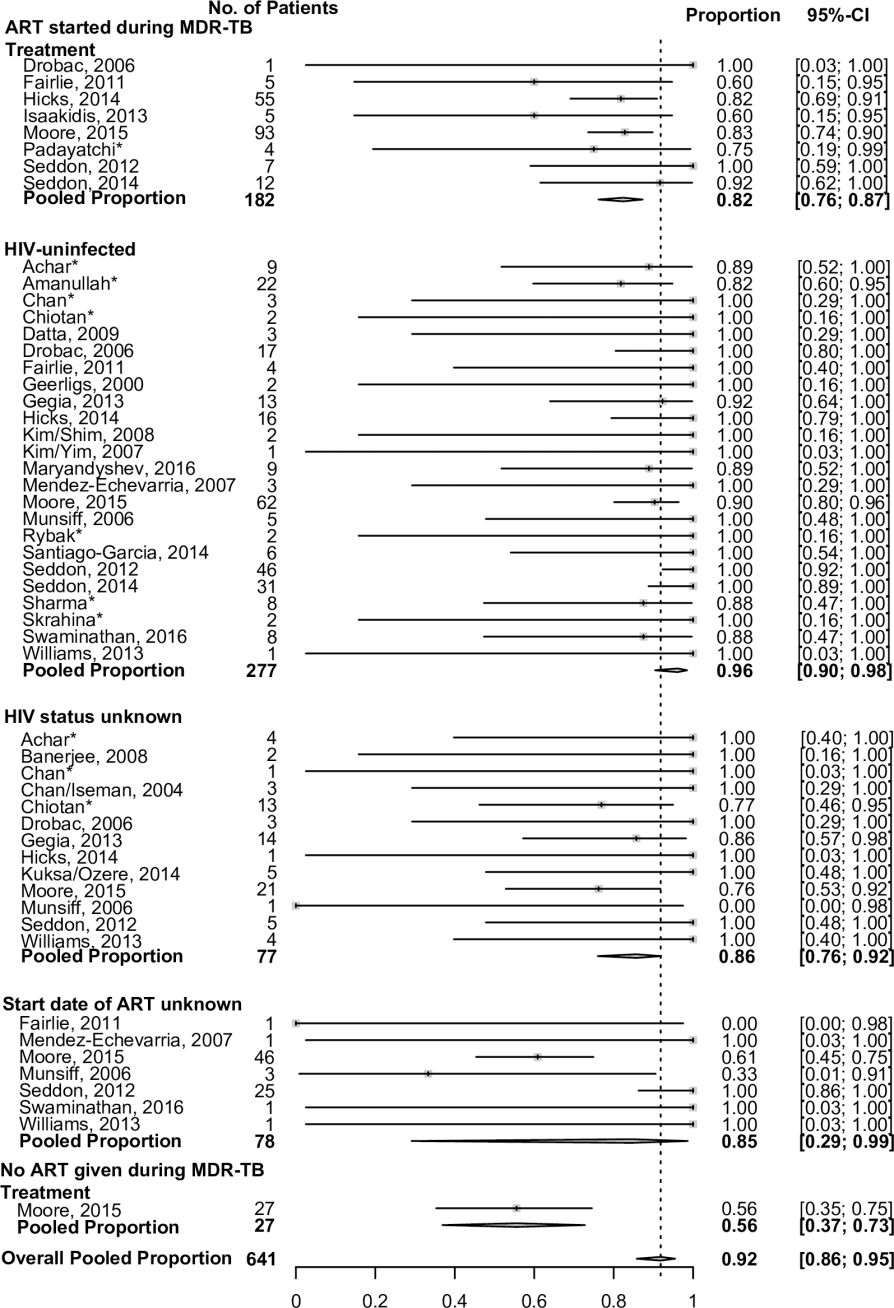
Proportion of bacteriologically confirmed MDR-TB patients achieving successful treatment outcomes, stratified by HIV infection and HIV ART. Data are stratified by HIV and ART status and by site. The 95% CIs were estimated using exact (Clopper-Pearson) method. Children from countries with very low HIV prevalence who did not have an HIV test were assumed not to be infected with HIV following consultation with site investigators. *Unpublished data. ART, antiretroviral treatment; MDR-TB, multidrug-resistant tuberculosis.

TB treatment outcomes are summarized in Table 3. Overall, 764 of 975 (78%) had a successful treatment outcome, with successful treatment outcomes in 548/731 (75%) of bacteriologically confirmed children and 216/244 (89%) in clinically diagnosed children. Treatment success rates did not differ among those children with pre-XDR, although numbers were small. Across all sites, most children had successful treatment outcomes, particularly those with a clinical diagnosis (Fig 3). There was considerable heterogeneity between sites with regard to treatment outcomes among confirmed cases, and less heterogeneity among clinical cases. Treatment outcomes among children infected with HIV assessed by the time of initiation of ART relative to MDR-TB therapy initiation are shown in Table 4. These data are presented by site in Fig 4. Treatment outcomes were worse in children infected with HIV who were never on any ART during their MDR-TB therapy, compared to children who received ART (either started before or concurrent with MDR-TB treatment). Among children with confirmed MDR-TB, treatment was successful in 56% (15/27) of children infected with HIV who did not receive any ART during MDR-TB therapy, compared to 82% (149/182) in children who received ART during MDR-TB therapy. Among children not infected with HIV, 93% with confirmed MDR-TB had successful treatment outcomes.

**Table 3:** Summary of treatment outcomes for children treated for MDR-TB.

MDR-TB treatment outcome	Bacteriologically confirmed MDR-TB *n* = 731 (%)	Clinically diagnosed MDR-TB *n* = 244 (%)	Absolute difference (95% CI)^a^	*p*-value	All children *n* = 975 (%)
Cured/completed treatment	548 (75)	216 (89)	14% (8%–19%)	<0.001	764 (78)
Death	77 (11)	8 (3)	8% (4%–11%)	<0.001	85 (9)
Failed treatment	16 (2)	0	2% (0%–4%)	0.044	16 (2)
Lost to follow-up/not evaluated	90 (12)	20 (8)	4% (0%–9%)	0.100	110 (11)

^a^The overall chi-squared *p*-value treatment outcomes between clinically diagnosed and bacteriologically confirmed cohorts is <0.001.

Abbreviation: MDR-TB, multidrug-resistant tuberculosis.

**Table 4:** Comparing treatment success rates among children with MDR-TB infected with HIV, by timing of initiation of ART and by HIV status^a^.

Characteristics	No ART during MDR-TB treatment, number treatment success^a^/*N* (%)	ART during MDR-TB treatment, number treatment success^a^/*N* (%)^b^	Absolute difference in proportion (ART versus No ART) (95% CI)	No data on ART, number treatment success/*N* (%)	Absolute difference in proportion (ART versus No data on ART) (95% CI)	*p*-value	All children infected with HIV, number treatment success^a^/*N* (%)	All children not infected with HIV, number treatment success^a^/*N* (%)	Absolute difference in proportion (HIV+ versus HIV−) (95% CI)
Bacteriologically confirmed MDR-TB	15/27 (56)	149/182 (82)	26% (5%–48%)	57/78 (73)	9% (−3%–21%)	0.006	221/287 (77)	291/312 (93)	16% (10%–22%)
Clinically diagnosed MDR-TB, number treatment success/N (%)	1/4 (25)	21/21 (100)	75% (18%–100%)	3/5 (60)	40% (−15%–95%)	<0.001	25/30 (83)	180/183 (98)	15% (0%–30%)

^a^Treatment success is defined as cured or completed treatment, lost to follow-up excluded. The overall chi-squared *p*-values comparing treatment success rate by ART status among bacteriologically confirmed and clinically diagnosed children infected with HIV, respectively, are *p* = 0.006 and *p* < 0.001.

^b^Either on ART before beginning MDR-TB treatment or ART started during MDR-TB treatment.

Abbreviations: ART, antiretroviral treatment; MDR-TB, multidrug-resistant tuberculosis.

In multivariable analysis, among children with confirmed MDR-TB, HIV infection (adjusted odds ratio [aOR] 0.3, 95% CI 0.2–0.6) and malnutrition (aOR 0.3, 95% CI 0.2–0.6) were associated with reduced odds of successful treatment outcome versus treatment failure or death (Table 5). Malnutrition was an independent risk factor from HIV infection for poor treatment outcome. This analysis did not adjust for the regimen used or individual drugs used. Among children with clinically diagnosed MDR-TB, no assessed covariates predicted treatment outcome.

**Table 5:** Clinical variables associated with treatment outcome in children with MDR-TB: *N* = 975.

Characteristics	Treatment success versus Failure/Death^a^					
	Bacteriologically confirmed *n* = 641^b^			Clinically diagnosed *n* = 224^b^		
Clinical variable	Adjusted OR	95% CI	*p*-value	Adjusted OR	95% CI	*p*-value
Age ≥5 years	1.36	0.78–2.37	0.191	9.64	0.53–176.98	0.969
Male	0.72	0.44–1.18	0.321	0.63	0.04–11.22	0.267
**Infected with HIV**^**e**^	**0.30**	**0.15–0.63**	<0.001	0.49	0.02–14.97	<0.001
Severe disease on chest radiography	0.66	0.32–1.34	0.001	0.07	<0.01–5.76	0.042
**Malnutrition**^**c**^^,^ ^**e**^	**0.33**	**0.19–0.60**	<0.001	0.01	<0.01–1.92	<0.001
Severe extrapulmonary disease^d^	0.60	0.32–1.13	0.026	0.09	<0.01–2.68	0.013

^a^Estimated using random effects models (random intercept and slope) with quadrature approximation. Missing values for sex, HIV, and severe extrapulmonary disease, severe disease on chest radiography, and malnutrition variables were imputed using the mean value of each variable for each site.

^b^Lost to follow-up was excluded from analysis.

^c^Malnutrition is defined as being underweight or malnourished by clinical diagnosis, having nutritional edema, or having low weight for age (weight-for-age-adjusted z-score of <−3).

^d^Severe extrapulmonary disease is defined as meningitis, miliary, abdominal, osteoarticlar, or

disseminated disease not otherwise specified.

^e^Bolded results met the prespecified criteria for statistical significance.

Abbreviations: MDR-TB, multidrug-resistant tuberculosis; OR, odds ratio.

### Treatment outcomes and specific drug therapy

We also conducted analyses to assess estimates of the association between individual antituberculosis medications, used as part of a multidrug regimen, and treatment success (Table 6) to inform the WHO treatment guideline process. This analysis was restricted to patients with bacteriologically confirmed MDR-TB. Although children who were lost to follow-up were excluded from the analysis, among children with confirmed MDR-TB, there were no differences in the following key variables between children who were lost to follow-up versus those with a known outcome: age, HIV status, sex, the presence of severe TB or severe extrapulmonary TB, previous TB treatment, or nutritional status (S4 Table). In children with confirmed MDR-TB, the second-line injectable agents and high-dose isoniazid (15–20 mg/kg/day) were associated with treatment success. Ninety-eight percent (130/133) of these children treated with high-dose isoniazid were from South Africa. When the association of individual drugs with treatment success was examined in South African data only, the CI widened but the point estimate remained the same (S5 Table). Among clinically diagnosed children, multivariable models were too unstable to provide reliable estimates.

**Table 6:** Summary of association of use of individual drugs with treatment success in children treated for confirmed MDR-TB (*n* = 641)^a^^,^^b^^,^^c^^,^^d^.

Drug used	*N* (%)	aOR^e^	95% CI	*p*-value
Pyrazinamide	599 (93)	1.63^g^	(0.41–6.56)	0.484
**Second-line injectable agents**^**f**^^,^ ^**j**^	**584 (91)**	**2.94**^g^	**(1.05–8.28)**	**0.041**
Ethionamide/ prothionamide	590 (92)	2.19	(0.42–11.54)	0.332
Cycloserine/ terizidone	356 (56)	1.66^h^	(0.91–3.05)	0.104
Clofazimine	23 (4)	0.55	(0.02–19.20)	0.714
**High-dose isoniazid**^**j**^	**133 (21)**	**5.86**^g^	**(1.68–20.51)**	**0.007**
Para-aminosalicylic acid	147 (23)	0.70^i^	(0.25–1.96)	0.483
Clarithromycin	32 (5)	0.29^i^	(0.05–1.53)	0.132

Treatment success was compared to Failure/Death by drug use.

^a^Adjusted estimates for the clinically diagnosed children were not possible due to very low rates of failure.

^b^Lost to follow-up was excluded from analysis.

^c^All random effects (random intercept and random slope) models used maximum likelihood estimation with quadrature approximation and were specified with an unstructured variance–covariance matrix parameterized through its Cholesky root unless otherwise stated.

^d^Too few children were treated with late-generation fluoroquinolones, carbapenems, and linezolid to be analyzed. No children in these cohorts were treated with bedaquiline or delamanid.

^e^aOR, for use of drug, with nonuse as reference category. Adjusted for age, sex, HIV infection, malnutrition, severity of disease on chest radiograph, and severity of extrapulmonary disease.

^f^Second-line injectable agents are amikacin, kanamycin, and capreomycin.

^g^Random-slope only model without random intercept, specified with standard variance components.

^h^Random-intercept only model without random slope, specified with standard variance components.

^i^Model specified with standard variance components (not unstructured).

^j^Bolded results met the prespecified criteria for statistical significance.

Abbreviations: aOR, adjusted odds ratio; MDR-TB, multidrug-resistant tuberculosis.

Among the clinically diagnosed children (who tended to have less severe disease on chest radiograph and lower rates of malnutrition and HIV), 60 (25%) received no injectable agent or less than 1 month of injectable agent. Fifty (83%) of these children had successful treatment outcomes.

Later generation fluoroquinolones were defined as moxifloxacin, gatifloxacin, sparfloxacin, or high-dose levofloxacin. Only 63/885 (7%) children in total were treated with moxifloxacin (44/641 [7%] in the confirmed and 19/244 [9%] in the clinically diagnosed groups). One child with confirmed disease received both gatifloxacin and sparfloxacin. Information on the dose of levofloxacin was available in only 93/885 (11%) of children. This infrequent use precluded useful analysis of the clinical impact of fluoroquinolones on treatment outcome.

## Discussion

This systematic review and IPD meta-analysis of pediatric MDR-TB represents a global collaborative effort of the pediatric TB community. It has for the first time, to our knowledge, generated a data set of children treated for MDR-TB in multiple countries, which has informed the revised 2016 WHO MDR-TB treatment guidelines [4].

A striking finding is the high proportion (78%) of successful treatment outcomes amongst all children, with 75% success in children with confirmed MDR-TB and 89% in those with clinically diagnosed disease. These good outcomes are despite the high prevalence of comorbidities (37% infected with HIV; 34% malnourished), severe pulmonary (49%) and extrapulmonary (13%) TB, and the abscence of the newer, safer, and more effective drugs. A previous systematic review, without IPD, reported a similar proportion of children with MDR-TB successfully treated (82%) [6]. These outcomes are considerably better than the treatment success rates (54%) reported in adults [9].

The association of HIV infection and malnutrition with reduced odds of successful TB treatment is consistent with the published literature [24,40]. Children infected with HIV but not receiving ART during their MDR-TB treatment were less likely to have successful treatment compared to children who were on ART at some point during their MDR-TB treatment episode. The 2016 WHO ART guidelines recommend that ART should be started as soon as possible and at least within 8 weeks of starting TB treatment in all TB patients infected with HIV, irrespective of CD4 count, with the possible exception of TB meningitis [41–44]. All data on children infected with HIV but not receiving ART came from a single study from South Africa (Fig 4) [21]; the data were collected from several public clinics throughout South Africa and are representative of the care children were receiving. Although the data all coming from a single study is a limitation, the finding that the lack of ART is associated with worse outcomes in people coinfected with TB and HIV has been well established [41, 45]. Our data support the importance of ART in children with TB who are infected with HIV, and highlight the need to aggressively treat children infected with HIV for both TB and HIV and to include nutritional support as part of standard care.

In this data set, children infected with HIV were more likely to have confirmed MDR-TB, despite reports of higher rates of paucibacillary TB disease in adults infected with HIV. This may be because the majority (95%) of children infected with HIV were from South Africa, where diagnostic tools and approaches may differ. There may also be hesitancy to start MDR-TB treatment in addition to ART; thus, treatment delays may lead to children having more severe TB disease, resulting in more bacteriologically confirmed disease. Children infected with HIV also tended to be older, as prevention of mother to child transmission (PMTCT) initiatives were rolling out during the study period in South Africa; this may have contributed to more children infected with HIV having a confirmed diagnosis.

A prominent finding was the benefit of high-dose (15–20 mg/kg/day) isoniazid. Twenty-one percent of children with confirmed MDR-TB received high-dose isoniazid; however, the majority of these children were from Cape Town. Despite attempts to statistically control for site-specific effect, it is possible that other unmeasured site-related effects, including the quality of clinical care, could have contributed to this finding. However, the fact that the point estimate for the benefit of high-dose isoniazid remained unchanged when South African data were examined demonstrates that this benefit remains, despite possible site-specific effect. When high-dose isoniazid is considered as part of MDR-TB treatment but the mutation conferring isoniazid resistance is unknown, the local epidemiology of isoniazid resistance mutations should be considered (high-level isoniazid resistance is conferred by the *katG* mutation and lower level resistance by the i*nhA* mutations) [46]. High-dose isoniazid can overcome an i*nhA* mutation’s low-level isoniazid resistance but is unlikely to overcome the high-level isoniazid resistance due to a *katG* gene mutation [47]. The Western Cape Province, South Africa (which includes Cape Town), has high rates of *inhA* mutations (61% of the isoniazid resistant mutations among pediatric patients [46]), which may have contributed to this high-dose isoniazid treatment benefit. However, none of the children underwent testing for isoniazid mutation and were therefore treated in a standard manner at the time the data were generated. Despite these caveats, our observations are consistent with other reports that suggest that high-dose isoniazid may have an important role in MDR-TB treatment as a component of shorter treatment regimens and as an add-on agent to the longer MDR-TB treatment regimens [1,48–50].

Among children with confirmed MDR-TB, the use of second-line injectable agents was associated with successful treatment versus failure/death. Although we found improved outcomes in children with confirmed disease when treated with injectable agents, we saw high levels of treatment success in children with clinically diagnosed MDR-TB who received no second-line injectable agents or who had received these parenteral antibiotics for less than 1 month. Eighty-three percent (50/60) of the children with clinically diagnosed disease (who tended to have less severe disease, with lower rates of HIV and malnutrition and less severe disease on chest radiography) who received no or less than 1 month’s treatment with a second-line injectable agent had successful treatment outcomes. These data need to be interpreted with caution and the use of second-line injectable agents in children needs more study. We did not collect data on the reason for using an injectable-free regimen, but possibilities include less severe disease, the presence of resistance to an injectable agent (although not all sites had access to extended drug-susceptibility testing), and perceived and actual injectable-associated adverse events.

Nonetheless, our findings lend support to not using second-line injectable agents in children with non-severe MDR-TB [6,18,51]. A previous study found hearing loss in 24% of children treated for MDR-TB with a second-line injectable agent [51]. Hearing loss can be devastating in children, impacting language acquisition and schooling, and administration of injectables is painful and resource intensive, frequently requiring daily visits to healthcare facilities. Avoiding second-line injectable agents should also be seen in the context of current and future MDR-TB treatment options. The new and repurposed MDR-TB drugs—bedaquiline, delamanid, linezolid, late-generation fluoroquinolones—were not used enough to be evaluated, but emerging evidence suggests they could be as effective and safer than the injectable agents [5,52].

Our results were used to inform the revision of the 2016 WHO MDR-TB treatment guidelines, which state that the harms associated with second-line injectable agents may outweigh potential benefits, and therefore injectable agents may be excluded from the treatment regimens of children with non-severe forms of MDR-TB disease [4].

A strength of our study was the inclusion of children with both bacteriologically confirmed and clinically diagnosed TB. Because of the paucibacillary nature of pediatric TB and the challenges of obtaining clinical samples, approximately 60%–70% of children treated globally for TB will be clinically diagnosed [25,35]. The inclusion of clinically diagnosed children makes our findings relevant to a broader range of children treated for MDR-TB, even if the proportion of children with clinically diagnosed MDR-TB (25%) was lower in our study than global values cited elsewhere. Notably, the low proportion of children in this study with clinically diagnosed MDR-TB may indicate potentially more severe pulmonary disease in our cohort, because bacteriological yield correlates with chest radiographic findings in children with TB [36]. The proportion of patients with bacteriologically versus clinically diagnosed TB differed by site (Fig 3). The reasons for this are likely varied; in some places, healthcare workers are reluctant to treat MDR-TB without bacteriological confirmation, and in some countries it is national policy that a bacteriological diagnosis be made in order for a child to be treated for MDR-TB, thereby excluding children with a clinical diagnosis from treatment. Some sites are MDR-TB specialty sites, so these may be better equipped to obtain samples and complete laboratory testing. Additionally, by the time children were evaluated at these tertiary centers, many probably had more advanced disease, which is more likely to be bacteriologically confirmed. Age may also explain some of our cohort’s attributes: some clinicians are less comfortable diagnosing TB in younger children, in whom the diagnosis is less likely to be confirmed and symptoms are frequently nonspecific. There are also likely publication biases involved here, whereby studies may have been more likely to have been published if they included more bacteriologically confirmed cases. However, in an attempt to combat this publication bias, we actively sought out unpublished data.

A criticism of studies that include clinically diagnosed children is that some of these children may not have MDR-TB and so treatment outcomes may not reflect true MDR-TB disease. In this study, among children with a clinical diagnosis, 67% had clinical TB with a known MDR-TB source case, and another 6% had failure of first-line therapy despite good adherence. Given that studies have shown that children are most likely to share the TB strain of their household contact, it is likely that the majority of children in this cohort did in fact have MDR-TB [37–39].

### Limitations

The most significant limitation of this study is that the estimated treatment effects of individual drugs should be interpreted with caution because the analysis could not isolate the benefit of each drug. We could only compare regimens with and without each drug. If particular drugs were used or not used together—because of disease severity or resistance profiles or toxicity—there is potential for confounding by indication that would produce biased effect estimates for each drug. Also, treatment decisions were based on the clinician’s perception of illness, with resulting potential for bias.

Furthermore, we were not able to compare specific regimens given the large variation in drugs used and treatment duration as part of individualized treatment. All studies included were retrospective or prospective observational cohort studies; there were no clinical trials. However, given that the study was an IPD, some adjustments could be made for covariates at the individual level, which is not possible in study-level meta-analyses. This worked to mitigate the risk of confounding and other potential biases. The sample size was modest compared to adult IPD data sets, and estimates were frequently imprecise, while some associations were not estimated because of limited data. There were no data available yet on the novel drugs, delamanid and bedaquiline, and data on linezolid were insufficient to include in this analysis. Data on late-generation fluoroquinolones and clofazimine were sparse. Children who were lost to follow-up and not evaluated (11% overall) were not included in this analysis, because their treatment outcome was unknown. Relapse was also not evaluated, as data on outcomes after completing antituberculosis treatment were rarely reported. This could have resulted in overestimation of favorable treatment outcome.

A further limitation is that, apart from DST for isoniazid and rifampicin, data about a strain’s susceptibility to other drugs (especially the fluoroquinolones and second-line injectable agents) were frequently not available for analysis; therefore, we could not assess the association between combinations of likely effective drugs (based on DST results) and outcomes. This may have also led to an underestimate of the benefit of drugs to which the strain was susceptible. Additionally, data on adverse events were incompletely reported and could not be included in the analysis. This scarcity of the reporting of adverse events also contributed to us being unable to distinguish treatment failure due to lack of efficacy from treatment failure due to drug intolerance.

Data were limited regarding the duration of posttreatment follow-up or posttreatment outcomes; therefore, we could not evaluate recurrence, relapse, or the effect of total duration of treatment on MDR-TB outcomes. Data on history of previous TB treatment were sparse and could not be analyzed.

All studies used consecutive sampling of children enrolled in MDR-TB treatment, which should have minimized selection bias. However, it is possible that the diagnosis of MDR-TB could have been missed in some children due to passive case finding at most study sites and general difficulties in diagnosing TB and MDR-TB in children. Children in whom the diagnosis may have been missed, often children with clinically diagnosed TB, who would usually have less severe, more paucibacillary disease, might have had different clinical characteristics and different outcomes than those included in the selected studies and therefore may have better treatment outcomes if they had in fact been enrolled. Thus, the likelihood of selection bias cannot be ruled out.

An additional limitation of this study was that the literature published after October 2014 was not included. In order to determine what effect this may have on our findings, we did a search of the pediatric MDR-TB literature from October 2014 to the present. The studies were reviewed, either full studies or abstracts, and details of studies that had the potential to be included in our study are included in S6 Table. As per the reasons listed in S6 Table, it is unlikely that including these in our study would have changed our findings.

### Conclusions

Children treated for MDR-TB have good treatment outcomes—even those with comorbidities and severe disease. That children tend to have much better treatment outcomes than adults raises the possibility that children may be specifically suited to do well with shorter, less intense treatment regimens. This deserves further study. Our study provides evidence that a substantial proportion of children with clinically diagnosed MDR-TB treated without second-line injectable agents have successful outcomes and that high-dose isoniazid may improve treatment success in some settings. Given the limitations of the current study, these findings should be interpreted cautiously but deserve further evaluation in subsequent studies. The high mortality and low treatment success rates among children infected with HIV who were not on ART during TB treatment demonstrates the importance of testing children with TB for HIV and initiating ART as soon as possible.

Data regarding the treatment outcomes and pharmacokinetic and safety profiles of bedaquiline, delamanid, later generation fluoroquinolones, linezolid, and clofazimine in children are urgently needed in children with MDR-TB, including in children infected with HIV. Future research is also needed on pediatric outcomes involving the shorter MDR-TB regimen [49,50]. Consensus on standardized reporting of demographic, clinical, and treatment characteristics for childhood MDR-TB would enhance the value of observational data and improve the comparability of results in published literature. It is notable that after extensive efforts to identify all possible pediatric cohorts, including unpublished cohorts, over a period of 24 years, fewer than 1,000 children with MDR-TB could be included in this analysis. Given that as many as 32,000 children develop MDR-TB each year [2], this represents a small fraction of children with MDR-TB. This paucity of literature highlights the fact that children with MDR-TB have been largely disregarded and underreported and that urgent attention needs to be paid to diagnose, treat, and report on this neglected population.

## Supporting information

S1 Table Search strategies for Pubmed and Embase. (DOCX)

S2 Table Newcastle-Ottawa approach for grading quality cohort studies used in this review. (DOCX)

S3 Table Overview of included studies and cohorts. (DOCX)

S4 Table Key clinical variables of children lost to follow-up versus those with known treatment outcomes. (DOCX)

S5 Table Summary of association of use of individual drugs with treatment success in children treated for confirmed multidrug-resistant tuberculosis in South African study sites. (DOCX)

S6 Table Overview of recent studies that may have been eligible for inclusion in this manuscript from October 2014 to present. (DOCX)

S1 Text PRISMA checklist. (DOC)

S2 Text Study protocol. (DOCX)

## Abbreviations

ADR: adverse drug reaction
AFB: acid-fast bacilli
aOR: adjusted odds ratio
ART: antiretroviral treatment
CI: confidence interval
DST: drug susceptibility test
ECDC: European Centre for Disease Prevention and Control
IPD: individual patient data
LILACS: Latin American and Caribbean Health Sciences Literature
MDR-TB: multidrug-resistant tuberculosis
MeSH: Medical Subject Headings
MSF: Médecins Sans Frontières
MTB: *Mycobacterium tuberculosis*
NIH: National Institutes of Health
PMTCT: prevention of mother to child transmission
pre-XDR-TB: pre-extensively drug-resistant tuberculosis
TB: tuberculosis
US CDC: United States Centers for Disease Control and Prevention
WHO: World Health Organization
XDR-TB: extensively drug-resistant tuberculosis
