# Supplementary material for: Treatment and outcomes in children with multidrug-resistant tuberculosis: A systematic review and individual patient data meta-analysis
Source: PLoS Med. 2018 Jul 11;15(7):e1002591. doi: 10.1371/journal.pmed.1002591 (PMC6040687; doi:10.1371/journal.pmed.1002591)
Supplement: S1 Table — (DOCX) [file pmed.1002591.s001.docx]

S1 Table. Search strategies for Pubmed and Embase

|  | Database: PubMed; Search date: 01-October 2014 |  |
| --- | --- | --- |
| Search | Query | Items found |
| [#25](http://www.ncbi.nlm.nih.gov/pubmed) | Search ((#22 AND #23) NOT (animals[mh] NOT humans[mh])) | [1653](http://www.ncbi.nlm.nih.gov/pubmed/?cmd=HistorySearch&querykey=25) |
| [#24](http://www.ncbi.nlm.nih.gov/pubmed) | Search (#22 AND #23) | [1653](http://www.ncbi.nlm.nih.gov/pubmed/?cmd=HistorySearch&querykey=24) |
| [#23](http://www.ncbi.nlm.nih.gov/pubmed) | Search (infant[mh] OR infant[tiab] OR infants[tiab] OR infancy[tiab] OR toddler*[tiab] OR preterm*[tiab] OR prematur*[tiab] OR postmatur*[tiab] OR baby[tiab] OR babies[tiab] OR neonat*[tiab] OR newborn[tiab] OR preschool*[tiab] OR pre-school*[tiab] OR child[mh] OR child*[tiab] OR kindergar*[tiab] OR pupil*[tiab] OR schoolchild*[tiab] OR teen*[tiab] OR youth[tiab] OR youths[tiab] OR youngster*[tiab] OR young person*[tiab] OR young people[tiab] OR minors[mh] OR minors[tiab] OR puberty[mh] OR puberty[tiab] OR pubescen*[tiab] OR prepubescen*[tiab] OR paediatric*[tiab] OR pediatric*[tiab] OR peadiatric*[tiab] OR schools[mh:noexp] OR school*[tiab] OR kid[tiab] OR kids[tiab] OR boy*[tiab] OR girl*[tiab] OR creche*[tiab] OR highschool*[tiab] OR juvenil*[tiab] OR adolescent[mh] OR adolescen*[tiab] OR under ag*[tiab] OR underag*[tiab]) | [3521601](http://www.ncbi.nlm.nih.gov/pubmed/?cmd=HistorySearch&querykey=23) |
| [#22](http://www.ncbi.nlm.nih.gov/pubmed) | Search (tuberculosis, multidrug-resistant[mh] OR multidrug resistant tuberculosis[tiab] OR drug resistant tuberculosis[tiab] OR multiple drug resistant tuberculosis[tiab] OR MDR tuberculosis[tiab] OR MDR TB[tiab] OR MDRTB[tiab] OR ((drug resistance[tiab] OR multidrug resistance[tiab] OR multiple drug resistance[tiab] OR multiresistant[tiab] OR multi resistant[tiab]) AND (tuberculosis[tiab] OR TB[tiab])) | [8600](http://www.ncbi.nlm.nih.gov/pubmed/?cmd=HistorySearch&querykey=22) |

- [AND in builder](http://www.ncbi.nlm.nih.gov/pubmed)
- [OR in builder](http://www.ncbi.nlm.nih.gov/pubmed)
- [NOT in builder](http://www.ncbi.nlm.nih.gov/pubmed)
- [Delete from history](http://www.ncbi.nlm.nih.gov/pubmed)
- [Show search results](http://www.ncbi.nlm.nih.gov/pubmed)
- [Show search details](http://www.ncbi.nlm.nih.gov/pubmed)
- [AND in builder](http://www.ncbi.nlm.nih.gov/pubmed)
- [OR in builder](http://www.ncbi.nlm.nih.gov/pubmed)
- [NOT in builder](http://www.ncbi.nlm.nih.gov/pubmed)
- [Delete from history](http://www.ncbi.nlm.nih.gov/pubmed)
- [Show search results](http://www.ncbi.nlm.nih.gov/pubmed)
- [Show search details](http://www.ncbi.nlm.nih.gov/pubmed)
- [Save in My NCBI](http://www.ncbi.nlm.nih.gov/pubmed)
- [AND in builder](http://www.ncbi.nlm.nih.gov/pubmed)
- [OR in builder](http://www.ncbi.nlm.nih.gov/pubmed)
- [NOT in builder](http://www.ncbi.nlm.nih.gov/pubmed)
- [Show search results](http://www.ncbi.nlm.nih.gov/pubmed)
- [Save as a My NCBI Collection](http://www.ncbi.nlm.nih.gov/pubmed)

| Database: EMBASE; Search date: 01-October 2014 | | |
| --- | --- | --- |
| No. | Query | Results |
| #8 | #3 NOT #7 | 1837 |
| #7 | #4 NOT #6 | 5002895 |
| #6 | #4 AND #5 | 1303481 |
| #5 | 'human'/de OR 'normal human'/de OR 'human cell'/de | 15207023 |
| #4 | 'animal'/de OR 'animal experiment'/de OR 'invertebrate'/de OR 'animal tissue'/de OR 'animal cell'/de OR 'nonhuman'/de | 6306376 |
| #3 | #1 AND #2 | 1889 |
| #2 | 'infant'/exp OR infant:ab,ti OR infants:ab,ti OR infancy:ab,ti OR toddler*:ab,ti OR preterm*:ab,ti OR prematur*:ab,ti OR postmatur*:ab,ti OR baby:ab,ti OR babies:ab,ti OR neonat*:ab,ti OR newborn:ab,ti OR preschool*:ab,ti OR pre+school*:ab,ti OR 'child'/exp OR child*:ab,ti OR kindergar*:ab,ti OR pupil*:ab,ti OR schoolchild*:ab,ti OR teen*:ab,ti OR youth:ab,ti OR youths:ab,ti OR youngster*:ab,ti OR 'young person':ab,ti OR 'young persons':ab,ti OR 'young people':ab,ti OR 'minors'/exp OR minors:ab,ti OR 'puberty'/exp OR puberty:ab,ti OR pubescen*:ab,ti OR prepubescen*:ab,ti OR paediatric*:ab,ti OR pediatric*:ab,ti OR peadiatric*:ab,ti OR 'schools'/exp OR school*:ab,ti OR kid:ab,ti OR kids:ab,ti OR boy*:ab,ti OR girl*:ab,ti OR creche*:ab,ti OR highschool*:ab,ti OR 'juvenile'/exp OR juvenil*:ab,ti OR 'adolescent'/exp OR adolescen*:ab,ti OR (under NEXT/1 ag*):ab,ti OR underag*:ab,ti | 4649411 |
| #1 | 'multidrug resistant tuberculosis'/exp OR 'multidrug resistant tuberculosis':ab,ti OR 'drug resistant tuberculosis':ab,ti OR 'multiple drug resistant tuberculosis':ab,ti OR 'mdr tuberculosis':ab,ti OR 'mdr tb':ab,ti OR ('drug resistance':ab,ti OR 'multidrug resistance':ab,ti OR 'multiple drug resistance':ab,ti OR multiresistant:ab,ti AND (tuberculosis:ab,ti OR tb:ab,ti)) | 9249 |
