# Supplementary material for: Treatment and outcomes in children with multidrug-resistant tuberculosis: A systematic review and individual patient data meta-analysis
Source: PLoS Med. 2018 Jul 11;15(7):e1002591. doi: 10.1371/journal.pmed.1002591 (PMC6040687; doi:10.1371/journal.pmed.1002591)
Supplement: S2 Table — (DOCX) [file pmed.1002591.s002.docx]

**S2 Table.** Newcastle-Ottawa approach for grading quality cohort studies used in this review

**Group: Study :**

| **Assessment of quality of a cohort study – Newcastle Ottawa Scale** |  |
| --- | --- |
| **Selection** (tick one box in each section) |  |
| 1. Representativeness of the study cohorta) truly representative of the average, 0-14 year old, MDR patient initiated on MDR-TB treatment (bacteriologically confirmed or clinically diagnosed) ★b) somewhat representative of the average, MDR patient ★c) selected group of patients, e.g. only certain socio-economic groups/areasd) no description of the derivation of the cohort | 🞏  🞏  🞏  🞏 |
| 2. ~~Selection of the non intervention cohort~~~~a) drawn from the same community as the intervention cohort ★~~~~b) drawn from a different source~~~~c) no description of the derivation of the non intervention cohort~~ | 🞏  🞏  🞏 |
| 2. Ascertainment of interventiona) secure record (eg health care record) ★b) structured interview ★c) written self reportd) other / no description | 🞏  🞏  🞏  🞏 |
| 4. Demonstration that outcome of interest was not present at start of studya) yes ★b) no | 🞏  🞏 |
| Comparability (tick one or both boxes, as appropriate) |  |
| 1. Comparability of cohorts on the basis of the design or analysisa) study controls for age, HIV infection, ★b) study controls for any additional factors (bacteriological status, disease severity, nutritional status) ★ | 🞏  🞏 |
| Outcome (tick one box in each section) |  |
| 1. Assessment of outcomea) independent blind assessment ★b) record linkage ★c) self report **d) other / no description** | 🞏  🞏  🞏  🞏 |
| 2. Was follow up long enough for outcomes to occura) yes, if median duration of follow-up >= 18 month ★b) no, if median duration of follow-up < 18 months | 🞏  🞏 |
| 3. Adequacy of follow up of cohortsa) complete follow up: all subjects accounted for ★b) subjects lost to follow up unlikely to introduce bias: number lost <= 20%, ★ or description of those lost suggesting no different from those followedc) follow up rate < 80% (select an adequate %) and no description of those lostd) no statement | 🞏  🞏  🞏  🞏 |

NB Underlined text ‘customised’ for the intervention being reviewed

**NOS – CODING MANUAL FOR COHORT STUDIES**

***SELECTION***

1. **Representativeness of the Exposed Cohort (NB exposure = intervention)**

Item is assessing the representativeness of exposed individuals in the community, not the representativeness of the study sample from some general population. For example, subjects derived from groups likely to contain exposed people are likely to be representative of exposed individuals, while they are not representative of all people the community.

*Allocation of stars as per rating sheet*

1. **Selection of the Non-Exposed Cohort**

*Allocation of stars as per rating sheet*

1. **Ascertainment of Exposure**

*Allocation of stars as per rating sheet*

1. **Demonstration That Outcome of Interest Was Not Present at Start of Study**

In the case of mortality studies, outcome of interest is still the presence of a disease/ incident, rather than death. That is to say that a statement of no history of disease or incident earns a star.

***COMPARABILITY***

1. **Comparability of Cohorts on the Basis of the Design or Analysis**

Either exposed and non-exposed individuals must be matched in the design and/or confounders must be adjusted for in the analysis. Statements of no differences between groups or that differences were not statistically significant are not sufficient for establishing comparability. Note: If the relative risk for the exposure of interest is adjusted for the confounders listed, then the groups will be considered to be comparable on each variable used in the adjustment.

*A maximum of 2 stars can be allotted in this category.*

***OUTCOME***

1. **Assessment of Outcome**

For some outcomes, reference to the medical record is sufficient to satisfy the requirement for confirmation. This may not be adequate for other outcomes where reference to specific tests or measures would be required.

1. Independent or blind assessment stated in the paper, or confirmation of the outcome by reference to secure records (health records, etc.)
2. Record linkage (e.g. identified through ICD codes on database records)
3. Self-report (i.e. no reference to original health records or documented source to confirm the outcome)
4. No description.
5. **Was Follow-Up Long Enough for Outcomes to Occur**

An acceptable length of time should be decided before quality assessment begins.

1. **Adequacy of Follow Up of Cohorts**

This item assesses the follow-up of the exposed and non-exposed cohorts to ensure that losses are not related to either the exposure or the outcome.

*Allocation of stars as per rating sheet*

**Group: Chan 2004 Study : unpublished**

**Treatment and outcome analysis of 205 patients with multidrug resistant tuberculosis**

| **Assessment of quality of a cohort study – Newcastle Ottawa Scale** |  |
| --- | --- |
| **Selection** (tick one box in each section) |  |
| 1. Representativeness of the study cohorta) truly representative of the average, 0-14 year old, MDR patient initiated on MDR-TB treatment (bacteriologically confirmed or clinically diagnosed) ★b) somewhat representative of the average, MDR patient ★c) selected group of patients, e.g. only certain socio-economic groups/areasd) no description of the derivation of the cohort | 🞏  X  🞏  🞏 |
| 2. ~~Selection of the non intervention cohort~~~~a) drawn from the same community as the intervention cohort ★~~~~b) drawn from a different source~~~~c) no description of the derivation of the non intervention cohort~~ | 🞏  🞏  🞏 |
| 2. Ascertainment of interventiona) secure record (eg health care record) ★b) structured interview ★c) written self reportd) other / no description | X  🞏  🞏  🞏 |
| 4. Demonstration that outcome of interest was not present at start of studya) yes ★b) no | 🞏  🞏 |
| Comparability (tick one or both boxes, as appropriate) |  |
| 1. Comparability of cohorts on the basis of the design or analysisa) study controls for age, HIV infection ★b) study controls for any additional factors (bacteriological status, disease severity, nutritional status) ★ | 🞏  🞏 |
| Outcome (tick one box in each section) |  |
| 1. Assessment of outcomea) independent blind assessment ★b) record linkage ★c) self report **d) other / no description** | 🞏  🞏  🞏  X |
| 2. Was follow up long enough for outcomes to occura) yes, if median duration of follow-up >= 18 month ★b) no, if median duration of follow-up < 18 months | X  🞏 |
| 3. Adequacy of follow up of cohortsa) complete follow up: all subjects accounted for ★b) subjects lost to follow up unlikely to introduce bias: number lost <= 20%, ★ or description of those lost suggesting no different from those followedc) follow up rate < 80% (select an adequate %) and no description of those lostd) no statement | X  🞏  🞏  🞏 |

NB Underlined text ‘customised’ for the intervention being reviewed

**Group:** Datta **Study :** Multidrug-resistant and extensively drug resistant tuberculosis in Kashmir, India

| **Assessment of quality of a cohort study – Newcastle Ottawa Scale** |  |
| --- | --- |
| **Selection** (tick one box in each section) |  |
| 1. Representativeness of the study cohorta) truly representative of the average, 0-14 year old, MDR patient initiated on MDR-TB treatment (bacteriologically confirmed or clinically diagnosed) ★b) somewhat representative of the average, MDR patient ★c) selected group of patients, e.g. only certain socio-economic groups/areasd) no description of the derivation of the cohort | X  🞏  🞏  🞏 |
| 2. ~~Selection of the non intervention cohort~~~~a) drawn from the same community as the intervention cohort ★~~~~b) drawn from a different source~~~~c) no description of the derivation of the non intervention cohort~~ | 🞏  🞏  🞏 |
| 2. Ascertainment of interventiona) secure record (eg health care record) ★b) structured interview ★c) written self reportd) other / no description | X  🞏  🞏  🞏 |
| 4. Demonstration that outcome of interest was not present at start of studya) yes ★b) no | 🞏  X |
| Comparability (tick one or both boxes, as appropriate) |  |
| 1. Comparability of cohorts on the basis of the design or analysisa) study controls for age, HIV infection, ★b) study controls for any additional factors (bacteriological status, disease severity, nutritional status) ★ | X  🞏 |
| Outcome (tick one box in each section) |  |
| 1. Assessment of outcomea) independent blind assessment ★b) record linkage ★c) self report **d) other/no description** | X  🞏  🞏  🞏 |
| 2. Was follow up long enough for outcomes to occura) yes, if median duration of follow-up >= 18 month ★b) no, if median duration of follow-up < 18 months | 🞏  🞏 |
| 3. Adequacy of follow up of cohortsa) complete follow up: all subjects accounted for ★b) subjects lost to follow up unlikely to introduce bias: number lost <= 20%, ★ or description of those lost suggesting no different from those followedc) follow up rate < 80% (select an adequate %) and no description of those lostd) no statement | 🞏  X  🞏  🞏 |

NB Underlined text ‘customised’ for the intervention being reviewed

**Group:** Drobac **Study :** Community based therapy for children with multi-drug resistant tuberculosis

| **Assessment of quality of a cohort study – Newcastle Ottawa Scale** |  |
| --- | --- |
| **Selection** (tick one box in each section) |  |
| 1. Representativeness of the study cohorta) truly representative of the average, 0-14 year old, MDR patient initiated on MDR-TB treatment (bacteriologically confirmed or clinically diagnosed) ★b) somewhat representative of the average, MDR patient ★c) selected group of patients, e.g. only certain socio-economic groups/areasd) no description of the derivation of the cohort | X  🞏  🞏  🞏 |
| 2. ~~Selection of the non intervention cohort~~~~a) drawn from the same community as the intervention cohort ★~~~~b) drawn from a different source~~~~c) no description of the derivation of the non intervention cohort~~ | 🞏  🞏  🞏 |
| 2. Ascertainment of interventiona) secure record (eg health care record) ★b) structured interview ★c) written self reportd) other / no description | X  🞏  🞏  🞏 |
| 4. Demonstration that outcome of interest was not present at start of studya) yes ★b) no | 🞏  X |
| Comparability (tick one or both boxes, as appropriate) |  |
| 1. Comparability of cohorts on the basis of the design or analysisa) study controls for age, HIV infection, ★b) study controls for any additional factors (bacteriological status, disease severity, nutritional status) ★ | X  X |
| Outcome (tick one box in each section) |  |
| 1. Assessment of outcomea) independent blind assessment ★b) record linkage ★c) self report **d) other / no description** | X  🞏  🞏  🞏 |
| 2. Was follow up long enough for outcomes to occura) yes, if median duration of follow-up >= 18 month ★b) no, if median duration of follow-up < 18 months | X  🞏 |
| 3. Adequacy of follow up of cohortsa) complete follow up: all subjects accounted for ★b) subjects lost to follow up unlikely to introduce bias: number lost <= 20%, ★ or description of those lost suggesting no different from those followedc) follow up rate < 80% (select an adequate %) and no description of those lostd) no statement | X  🞏  🞏  🞏 |

NB Underlined text ‘customised’ for the intervention being reviewed

**Group:**Fairlie **Study :** High prevalence of childhood multidrug resistant tuberculosis in Johannesburg, South Africa: a cross sectional study

| **Assessment of quality of a cohort study – Newcastle Ottawa Scale** |  |
| --- | --- |
| **Selection** (tick one box in each section) |  |
| 1. Representativeness of the study cohorta) truly representative of the average, 0-14 year old, MDR patient initiated on MDR-TB treatment (bacteriologically confirmed or clinically diagnosed) ★b) somewhat representative of the average, MDR patient ★c) selected group of patients, e.g. only certain socio-economic groups/areasd) no description of the derivation of the cohort | X  🞏  🞏  🞏 |
| 2. ~~Selection of the non intervention cohort~~~~a) drawn from the same community as the intervention cohort ★~~~~b) drawn from a different source~~~~c) no description of the derivation of the non intervention cohort~~ | 🞏  🞏  🞏 |
| 2. Ascertainment of interventiona) secure record (eg health care record) ★b) structured interview ★c) written self reportd) other / no description | X  🞏  🞏  🞏 |
| 4. Demonstration that outcome of interest was not present at start of studya) yes ★b) no | 🞏  X |
| Comparability (tick one or both boxes, as appropriate) |  |
| 1. Comparability of cohorts on the basis of the design or analysisa) study controls for age, HIV infection, ★b) study controls for any additional factors (bacteriological status, disease severity, nutritional status) ★ | X  🞏 |
| Outcome (tick one box in each section) |  |
| 1. Assessment of outcomea) independent blind assessment ★b) record linkage ★c) self report **d) other / no description** | X  🞏  🞏  🞏 |
| 2. Was follow up long enough for outcomes to occura) yes, if median duration of follow-up >= 18 month ★b) no, if median duration of follow-up < 18 months | 🞏  🞏 |
| 3. Adequacy of follow up of cohortsa) complete follow up: all subjects accounted for ★b) subjects lost to follow up unlikely to introduce bias: number lost <= 20%, ★ or description of those lost suggesting no different from those followedc) follow up rate < 80% (select an adequate %) and no description of those lostd) no statement | 🞏  X  🞏  🞏 |

NB Underlined text ‘customised’ for the intervention being reviewed

**Group:** Geerligs **Study:** Multi-drug resistant tuberculosis: long-term treatment outcome in the Netherlands

| **Assessment of quality of a cohort study – Newcastle Ottawa Scale** |  |
| --- | --- |
| **Selection** (tick one box in each section) |  |
| 1. Representativeness of the intervention cohorta) truly representative of the average, 0-14, MDR patient bacteriologically or clinically confirmed ★b) somewhat representative of the average, MDR patient ★c) selected group of patients, e.g. only certain socio-economic groups/areasd) no description of the derivation of the cohort | 🞏  X  🞏  🞏 |
| 2. ~~Selection of the non intervention cohort~~~~a) drawn from the same community as the intervention cohort ★~~~~b) drawn from a different source~~~~c) no description of the derivation of the non intervention cohort~~ | 🞏  🞏  🞏 |
| 3. Ascertainment of interventiona) secure record (eg health care record) ★b) structured interview ★c) written self reportd) other / no description | X  🞏  🞏  🞏 |
| 4. Demonstration that outcome of interest was not present at start of studya) yes ★b) no | X  🞏 |
| Comparability (tick one or both boxes, as appropriate) |  |
| 1. Comparability of cohorts on the basis of the design or analysisa) study controls for age, bacteriological status, co-morbid conditions ★b) study controls for any additional factors (e.g. socio-economic status, education) ★ | X  🞏 |
| Outcome (tick one box in each section) |  |
| 1. Assessment of outcomea) independent blind assessment ★b) record linkage ★c) self report **d) other / no description** | 🞏  X  🞏  🞏 |
| 2. Was follow up long enough for outcomes to occura) yes, if median duration of follow-up >= 18 month ★b) no, if median duration of follow-up < 18 months | X  🞏 |
| 3. Adequacy of follow up of cohortsa) complete follow up: all subjects accounted for ★b) subjects lost to follow up unlikely to introduce bias: number lost <= 20%, ★ or description of those lost suggesting no different from those followedc) follow up rate < 80% (select an adequate %) and no description of those lostd) no statement | X  🞏  🞏  🞏 |

NB Underlined text ‘customised’ for the intervention being reviewed

**Group: Gegia Study:** Outcomes of children treated for tuberculosis with second-line medications in Georgia, 2009-2011

| **Assessment of quality of a cohort study – Newcastle Ottawa Scale** |  |
| --- | --- |
| **Selection** (tick one box in each section) |  |
| 1. Representativeness of the intervention cohorta) truly representative of the average, 0-14, MDR patient bacteriologically or clinically confirmed ★b) somewhat representative of the average, MDR patient ★c) selected group of patients, e.g. only certain socio-economic groups/areasd) no description of the derivation of the cohort | X  🞏  🞏  🞏 |
| 2. ~~Selection of the non intervention cohort~~~~a) drawn from the same community as the intervention cohort ★~~~~b) drawn from a different source~~~~c) no description of the derivation of the non intervention cohort~~ | 🞏  🞏  🞏 |
| 3. Ascertainment of interventiona) secure record (eg health care record) ★b) structured interview ★c) written self reportd) other / no description | X  🞏  🞏  🞏 |
| 4. Demonstration that outcome of interest was not present at start of studya) yes ★b) no | X  🞏 |
| Comparability (tick one or both boxes, as appropriate) |  |
| 1. Comparability of cohorts on the basis of the design or analysisa) study controls for age, bacteriological status, co-morbid conditions ★b) study controls for any additional factors (e.g. socio-economic status, education) ★ | X  🞏 |
| Outcome (tick one box in each section) |  |
| 1. Assessment of outcomea) independent blind assessment ★b) record linkage ★c) self report **d) other / no description** | 🞏  X  🞏  🞏 |
| 2. Was follow up long enough for outcomes to occura) yes, if median duration of follow-up >= 18 month ★b) no, if median duration of follow-up < 18 months (in the IPD dataset, only patients with long term outcome were included) | X  🞏 |
| 3. Adequacy of follow up of cohortsa) complete follow up: all subjects accounted for ★b) subjects lost to follow up unlikely to introduce bias: number lost <= 20%, ★ or description of those lost suggesting no different from those followedc) follow up rate < 80% (select an adequate %) and no description of those lostd) no statement | X  🞏  🞏  🞏 |

NB Underlined text ‘customised’ for the intervention being reviewed

**Group:** Granich **Study :** Multidrug resistance among persons with tuberculosis in California, 1994–2003

| **Assessment of quality of a cohort study – Newcastle Ottawa Scale** |  |
| --- | --- |
| **Selection** (tick one box in each section) |  |
| 1. Representativeness of the study cohorta) truly representative of the average, 0-14 year old, MDR patient initiated on MDR-TB treatment (bacteriologically confirmed or clinically diagnosed) ★b) somewhat representative of the average, MDR patient ★c) selected group of patients, e.g. only certain socio-economic groups/areasd) no description of the derivation of the cohort | X  🞏  🞏  🞏 |
| 2. ~~Selection of the non intervention cohort~~~~a) drawn from the same community as the intervention cohort ★~~~~b) drawn from a different source~~~~c) no description of the derivation of the non intervention cohort~~ | 🞏  🞏  🞏 |
| 2. Ascertainment of interventiona) secure record (eg health care record) ★b) structured interview ★c) written self reportd) other / no description | X  🞏  🞏  🞏 |
| 4. Demonstration that outcome of interest was not present at start of studya) yes ★b) no | 🞏  X |
| Comparability (tick one or both boxes, as appropriate) |  |
| 1. Comparability of cohorts on the basis of the design or analysisa) study controls for age, HIV infection, ★b) study controls for any additional factors (bacteriological status, disease severity, nutritional status) ★ | X  🞏 |
| Outcome (tick one box in each section) |  |
| 1. Assessment of outcomea) independent blind assessment ★b) record linkage ★c) self report **d) other / no description** | X  🞏  🞏  🞏 |
| 2. Was follow up long enough for outcomes to occura) yes, if median duration of follow-up >= 18 month ★b) no, if median duration of follow-up < 18 months | X  🞏 |
| 3. Adequacy of follow up of cohortsa) complete follow up: all subjects accounted for ★b) subjects lost to follow up unlikely to introduce bias: number lost <= 20%, ★ or description of those lost suggesting no different from those followedc) follow up rate < 80% (select an adequate %) and no description of those lostd) no statement | 🞏  🞏  X  🞏 |

NB Underlined text ‘customised’ for the intervention being reviewed

**Group:** Hicks **Study :** Malnutrition associated with unfavorable outcome among South African MDR-TB and HIV co-infected children

| **Assessment of quality of a cohort study – Newcastle Ottawa Scale** |  |
| --- | --- |
| **Selection** (tick one box in each section) |  |
| 1. Representativeness of the study cohorta) truly representative of the average, 0-14 year old, MDR patient initiated on MDR-TB treatment (bacteriologically confirmed or clinically diagnosed) ★b) somewhat representative of the average, MDR patient ★c) selected group of patients, e.g. only certain socio-economic groups/areasd) no description of the derivation of the cohort | X  🞏  🞏  🞏 |
| 2. ~~Selection of the non intervention cohort~~~~a) drawn from the same community as the intervention cohort ★~~~~b) drawn from a different source~~~~c) no description of the derivation of the non intervention cohort~~ | 🞏  🞏  🞏 |
| 2. Ascertainment of interventiona) secure record (eg health care record) ★b) structured interview ★c) written self reportd) other / no description | X  🞏  🞏  🞏 |
| 4. Demonstration that outcome of interest was not present at start of studya) yes ★b) no | 🞏  X |
| Comparability (tick one or both boxes, as appropriate) |  |
| 1. Comparability of cohorts on the basis of the design or analysisa) study controls for age, HIV infection, ★b) study controls for any additional factors (bacteriological status, disease severity, nutritional status) ★ | X  X |
| Outcome (tick one box in each section) |  |
| 1. Assessment of outcomea) independent blind assessment ★b) record linkage ★c) self report **d) other / no description** | X  🞏  🞏  🞏 |
| 2. Was follow up long enough for outcomes to occura) yes, if median duration of follow-up >= 18 month ★b) no, if median duration of follow-up < 18 months | X  🞏 |
| 3. Adequacy of follow up of cohortsa) complete follow up: all subjects accounted for ★b) subjects lost to follow up unlikely to introduce bias: number lost <= 20%, ★ or description of those lost suggesting no different from those followedc) follow up rate < 80% (select an adequate %) and no description of those lostd) no statement | 🞏  X  🞏  🞏 |

NB Underlined text ‘customised’ for the intervention being reviewed

**Group:** Isaakidis **Study :** Poor Outcomes in a Cohort of HIV-Infected Adolescents Undergoing Treatment for Multidrug-Resistant Tuberculosis in Mumbai, India

| **Assessment of quality of a cohort study – Newcastle Ottawa Scale** |  |
| --- | --- |
| **Selection** (tick one box in each section) |  |
| 1. Representativeness of the study cohorta) truly representative of the average, 0-14 year old, MDR patient initiated on MDR-TB treatment (bacteriologically confirmed or clinically diagnosed) ★b) somewhat representative of the average, MDR patient ★c) selected group of patients, e.g. only certain socio-economic groups/areasd) no description of the derivation of the cohort | 🞏  X  🞏  🞏 |
| 2. ~~Selection of the non intervention cohort~~~~a) drawn from the same community as the intervention cohort ★~~~~b) drawn from a different source~~~~c) no description of the derivation of the non intervention cohort~~ | 🞏  🞏  🞏 |
| 2. Ascertainment of interventiona) secure record (eg health care record) ★b) structured interview ★c) written self reportd) other / no description | X  🞏  🞏  🞏 |
| 4. Demonstration that outcome of interest was not present at start of studya) yes ★b) no | 🞏  X |
| Comparability (tick one or both boxes, as appropriate) |  |
| 1. Comparability of cohorts on the basis of the design or analysisa) study controls for age, HIV infection, ★b) study controls for any additional factors (bacteriological status, disease severity, nutritional status) ★ | X  🞏 |
| Outcome (tick one box in each section) |  |
| 1. Assessment of outcomea) independent blind assessment ★b) record linkage ★c) self report **d) other / no description** | X  🞏  🞏  🞏 |
| 2. Was follow up long enough for outcomes to occura) yes, if median duration of follow-up >= 18 month ★b) no, if median duration of follow-up < 18 months | X  🞏 |
| 3. Adequacy of follow up of cohortsa) complete follow up: all subjects accounted for ★b) subjects lost to follow up unlikely to introduce bias: number lost <= 20%, ★ or description of those lost suggesting no different from those followedc) follow up rate < 80% (select an adequate %) and no description of those lostd) no statement | X  🞏  🞏  🞏 |

NB Underlined text ‘customised’ for the intervention being reviewed

**Group: Kim/**Shim **Study :** Treatment Outcomes and Long-term Survival in Patients with Extensively Drug-resistant Tuberculosis

| **Assessment of quality of a cohort study – Newcastle Ottawa Scale** |  |
| --- | --- |
| **Selection** (tick one box in each section) |  |
| 1. Representativeness of the study cohorta) truly representative of the average, 0-14 year old, MDR patient initiated on MDR-TB treatment (bacteriologically confirmed or clinically diagnosed) ★b) somewhat representative of the average, MDR patient ★c) selected group of patients, e.g. only certain socio-economic groups/areasd) no description of the derivation of the cohort | 🞏  X  🞏  🞏 |
| 2. ~~Selection of the non intervention cohort~~~~a) drawn from the same community as the intervention cohort ★~~~~b) drawn from a different source~~~~c) no description of the derivation of the non intervention cohort~~ | 🞏  🞏  🞏 |
| 2. Ascertainment of interventiona) secure record (eg health care record) ★b) structured interview ★c) written self reportd) other / no description | X  🞏  🞏  🞏 |
| 4. Demonstration that outcome of interest was not present at start of studya) yes ★b) no | X  🞏 |
| Comparability (tick one or both boxes, as appropriate) |  |
| 1. Comparability of cohorts on the basis of the design or analysisa) study controls for age, HIV infection, ★b) study controls for any additional factors (bacteriological status, disease severity, nutritional status) ★ | X  🞏 |
| Outcome (tick one box in each section) |  |
| 1. Assessment of outcomea) independent blind assessment ★b) record linkage ★c) self report **d) other / no description** | 🞏  🞏  🞏  X |
| 2. Was follow up long enough for outcomes to occura) yes, if median duration of follow-up >= 18 month ★b) no, if median duration of follow-up < 18 months | X  🞏 |
| 3. Adequacy of follow up of cohortsa) complete follow up: all subjects accounted for ★b) subjects lost to follow up unlikely to introduce bias: number lost <= 20%, ★ or description of those lost suggesting no different from those followedc) follow up rate < 80% (select an adequate %) and no description of those lostd) no statement | 🞏  🞏  X  🞏 |

NB Underlined text ‘customised’ for the intervention being reviewed

**Group: Kim/**Yim **Study :** Impact of Extensive drug resistance on treatment outcomes in non-HIV infected patients with multi-drug resistant tuberculosis

| **Assessment of quality of a cohort study – Newcastle Ottawa Scale** |  |
| --- | --- |
| **Selection** (tick one box in each section) |  |
| 1. Representativeness of the study cohorta) truly representative of the average, 0-14 year old, MDR patient initiated on MDR-TB treatment (bacteriologically confirmed or clinically diagnosed) ★b) somewhat representative of the average, MDR patient ★c) selected group of patients, e.g. only certain socio-economic groups/areasd) no description of the derivation of the cohort | 🞏  X  🞏  🞏 |
| 2. ~~Selection of the non intervention cohort~~~~a) drawn from the same community as the intervention cohort ★~~~~b) drawn from a different source~~~~c) no description of the derivation of the non intervention cohort~~ | 🞏  🞏  🞏 |
| 2. Ascertainment of interventiona) secure record (eg health care record) ★b) structured interview ★c) written self reportd) other / no description | X  🞏  🞏  🞏 |
| 4. Demonstration that outcome of interest was not present at start of studya) yes ★b) no | 🞏  X |
| Comparability (tick one or both boxes, as appropriate) |  |
| 1. Comparability of cohorts on the basis of the design or analysisa) study controls for age, HIV infection, ★b) study controls for any additional factors (bacteriological status, disease severity, nutritional status) ★ | X  X |
| Outcome (tick one box in each section) |  |
| 1. Assessment of outcomea) independent blind assessment ★b) record linkage ★c) self report **d) other / no description** | X  🞏  🞏  🞏 |
| 2. Was follow up long enough for outcomes to occura) yes, if median duration of follow-up >= 18 month ★b) no, if median duration of follow-up < 18 months | X  🞏 |
| 3. Adequacy of follow up of cohortsa) complete follow up: all subjects accounted for ★b) subjects lost to follow up unlikely to introduce bias: number lost <= 20%, ★ or description of those lost suggesting no different from those followedc) follow up rate < 80% (select an adequate %) and no description of those lostd) no statement | 🞏  X  🞏  🞏 |

NB Underlined text ‘customised’ for the intervention being reviewed

**Group:** Kuksa **Study :** Multi and extensively drug resistant tuberculosis in Latvia: trends, characteristics and treatment outcomes

| **Assessment of quality of a cohort study – Newcastle Ottawa Scale** |  |
| --- | --- |
| **Selection** (tick one box in each section) |  |
| 1. Representativeness of the study cohorta) truly representative of the average, 0-14 year old, MDR patient initiated on MDR-TB treatment (bacteriologically confirmed or clinically diagnosed) ★b) somewhat representative of the average, MDR patient ★c) selected group of patients, e.g. only certain socio-economic groups/areasd) no description of the derivation of the cohort | X  🞏  🞏  🞏 |
| 2. ~~Selection of the non intervention cohort~~~~a) drawn from the same community as the intervention cohort ★~~~~b) drawn from a different source~~~~c) no description of the derivation of the non intervention cohort~~ | 🞏  🞏  🞏 |
| 2. Ascertainment of interventiona) secure record (eg health care record) ★b) structured interview ★c) written self reportd) other / no description | X  🞏  🞏  🞏 |
| 4. Demonstration that outcome of interest was not present at start of studya) yes ★b) no | 🞏  X |
| Comparability (tick one or both boxes, as appropriate) |  |
| 1. Comparability of cohorts on the basis of the design or analysisa) study controls for age, HIV infection, ★b) study controls for any additional factors (bacteriological status, disease severity, nutritional status) ★ | X  🞏 |
| Outcome (tick one box in each section) |  |
| 1. Assessment of outcomea) independent blind assessment ★b) record linkage ★c) self report **d) other / no description** | X  🞏  🞏  🞏 |
| 2. Was follow up long enough for outcomes to occura) yes, if median duration of follow-up >= 18 month ★b) no, if median duration of follow-up < 18 months | 🞏  🞏 |
| 3. Adequacy of follow up of cohortsa) complete follow up: all subjects accounted for ★b) subjects lost to follow up unlikely to introduce bias: number lost <= 20%, ★ or description of those lost suggesting no different from those followedc) follow up rate < 80% (select an adequate %) and no description of those lostd) no statement | 🞏  X  🞏  🞏 |

NB Underlined text ‘customised’ for the intervention being reviewed

**Group:** Méndez Echevarría **Study** : Multidrug resistant tuberculosis in the pediatric age group

| **Assessment of quality of a cohort study – Newcastle Ottawa Scale** |  |
| --- | --- |
| **Selection** (tick one box in each section) |  |
| 1. Representativeness of the study cohorta) truly representative of the average, 0-14 year old, MDR patient initiated on MDR-TB treatment (bacteriologically confirmed or clinically diagnosed) ★b) somewhat representative of the average, MDR patient ★c) selected group of patients, e.g. only certain socio-economic groups/areasd) no description of the derivation of the cohort | 🞏  X  🞏  🞏 |
| 2. ~~Selection of the non intervention cohort~~~~a) drawn from the same community as the intervention cohort ★~~~~b) drawn from a different source~~~~c) no description of the derivation of the non intervention cohort~~ | 🞏  🞏  🞏 |
| 2. Ascertainment of interventiona) secure record (eg health care record) ★b) structured interview ★c) written self reportd) other / no description | X  🞏  🞏  🞏 |
| 4. Demonstration that outcome of interest was not present at start of studya) yes ★b) no | X  🞏 |
| Comparability (tick one or both boxes, as appropriate) This is a case report series, so there are no controls |  |
| 1. Comparability of cohorts on the basis of the design or analysisa) study controls for age, HIV infection, ★b) study controls for any additional factors (bacteriological status, disease severity, nutritional status) ★ | 🞏  🞏 |
| Outcome (tick one box in each section) |  |
| 1. Assessment of outcomea) independent blind assessment ★b) record linkage ★c) self report **d) other / no description** | 🞏  X  🞏  🞏 |
| 2. Was follow up long enough for outcomes to occura) yes, if median duration of follow-up >= 18 month ★b) no, if median duration of follow-up < 18 months | X  🞏 |
| 3. Adequacy of follow up of cohortsa) complete follow up: all subjects accounted for ★b) subjects lost to follow up unlikely to introduce bias: number lost <= 20%, ★ or description of those lost suggesting no different from those followedc) follow up rate < 80% (select an adequate %) and no description of those lostd) no statement | X  🞏  🞏  🞏 |

NB Underlined text ‘customised’ for the intervention being reviewed

**Group:** Moore **Study :** Epidemiology of drug resistant tuberculosis among children and adolescents in South Africa, 2005-2010

| **Assessment of quality of a cohort study – Newcastle Ottawa Scale** |  |
| --- | --- |
| **Selection** (tick one box in each section) |  |
| 1. Representativeness of the study cohorta) truly representative of the average, 0-14 year old, MDR patient initiated on MDR-TB treatment (bacteriologically confirmed or clinically diagnosed) ★b) somewhat representative of the average, MDR patient ★c) selected group of patients, e.g. only certain socio-economic groups/areasd) no description of the derivation of the cohort | X  🞏  🞏  🞏 |
| 2. ~~Selection of the non intervention cohort~~~~a) drawn from the same community as the intervention cohort ★~~~~b) drawn from a different source~~~~c) no description of the derivation of the non intervention cohort~~ | 🞏  🞏  🞏 |
| 2. Ascertainment of interventiona) secure record (eg health care record) ★b) structured interview ★c) written self reportd) other / no description | X  🞏  🞏  🞏 |
| 4. Demonstration that outcome of interest was not present at start of studya) yes ★b) no | 🞏  X |
| Comparability (tick one or both boxes, as appropriate) |  |
| 1. Comparability of cohorts on the basis of the design or analysisa) study controls for age, HIV infection, ★b) study controls for any additional factors (bacteriological status, disease severity, nutritional status) ★ | X  X |
| Outcome (tick one box in each section) |  |
| 1. Assessment of outcomea) independent blind assessment ★b) record linkage ★c) self report **d) other / no description** | X  🞏  🞏  🞏 |
| 2. Was follow up long enough for outcomes to occura) yes, if median duration of follow-up >= 18 month ★b) no, if median duration of follow-up < 18 months | X  🞏 |
| 3. Adequacy of follow up of cohortsa) complete follow up: all subjects accounted for ★b) subjects lost to follow up unlikely to introduce bias: number lost <= 20%, ★ or description of those lost suggesting no different from those followedc) follow up rate < 80% (select an adequate %) and no description of those lostd) no statement | 🞏  X  🞏  🞏 |

NB Underlined text ‘customised’ for the intervention being reviewed

**Group:** Munsiff  **Study:** Public-private collaboration for multidrug-resistant tuberculosis control in New York City

| **Assessment of quality of a cohort study – Newcastle Ottawa Scale** |  |
| --- | --- |
| **Selection** (tick one box in each section) |  |
| 1. Representativeness of the study cohorta) truly representative of the average, 0-14 year old, MDR patient initiated on MDR-TB treatment (bacteriologically confirmed or clinically diagnosed) ★b) somewhat representative of the average, MDR patient ★c) selected group of patients, e.g. only certain socio-economic groups/areasd) no description of the derivation of the cohort | X  🞏  🞏  🞏 |
| 2. ~~Selection of the non intervention cohort~~~~a) drawn from the same community as the intervention cohort ★~~~~b) drawn from a different source~~~~c) no description of the derivation of the non intervention cohort~~ | 🞏  🞏  🞏 |
| 2. Ascertainment of interventiona) secure record (eg health care record) ★b) structured interview ★c) written self reportd) other / no description | X  🞏  🞏  🞏 |
| 4. Demonstration that outcome of interest was not present at start of studya) yes ★b) no | X  🞏 |
| Comparability (tick one or both boxes, as appropriate) |  |
| 1. Comparability of cohorts on the basis of the design or analysisa) study controls for age, HIV infection, ★b) study controls for any additional factors (bacteriological status, disease severity, nutritional status) ★ | X  🞏 |
| Outcome (tick one box in each section) |  |
| 1. Assessment of outcomea) independent blind assessment ★b) record linkage ★c) self report **d) other / no description** | 🞏  X  🞏  🞏 |
| 2. Was follow up long enough for outcomes to occura) yes, if median duration of follow-up >= 18 month ★b) no, if median duration of follow-up < 18 months | X  🞏 |
| 3. Adequacy of follow up of cohortsa) complete follow up: all subjects accounted for ★b) subjects lost to follow up unlikely to introduce bias: number lost <= 20%, ★ or description of those lost suggesting no different from those followedc) follow up rate < 80% (select an adequate %) and no description of those lostd) no statement | X  🞏  🞏  🞏 |

NB Underlined text ‘customised’ for the intervention being reviewed

**Group:** Santiago-Garcia **Study : Pediatric drug-resistant tuberculosis in Madrid: Family matters**

| **Assessment of quality of a cohort study – Newcastle Ottawa Scale** |  |
| --- | --- |
| **Selection** (tick one box in each section) |  |
| 1. Representativeness of the study cohorta) truly representative of the average, 0-14 year old, MDR patient initiated on MDR-TB treatment (bacteriologically confirmed or clinically diagnosed) ★b) somewhat representative of the average, MDR patient ★c) selected group of patients, e.g. only certain socio-economic groups/areasd) no description of the derivation of the cohort | 🞏  X  🞏  🞏 |
| 2. ~~Selection of the non intervention cohort~~~~a) drawn from the same community as the intervention cohort ★~~~~b) drawn from a different source~~~~c) no description of the derivation of the non intervention cohort~~ | 🞏  🞏  🞏 |
| 2. Ascertainment of interventiona) secure record (eg health care record) ★b) structured interview ★c) written self reportd) other / no description | X  🞏  🞏  🞏 |
| 4. Demonstration that outcome of interest was not present at start of studya) yes ★b) no | X  🞏 |
| Comparability (tick one or both boxes, as appropriate) |  |
| 1. Comparability of cohorts on the basis of the design or analysisa) study controls for age, HIV infection, ★b) study controls for any additional factors (bacteriological status, disease severity, nutritional status) ★ | X  X |
| Outcome (tick one box in each section) |  |
| 1. Assessment of outcomea) independent blind assessment ★b) record linkage ★c) self report **d) other / no description** | 🞏  🞏  🞏  X |
| 2. Was follow up long enough for outcomes to occura) yes, if median duration of follow-up >= 18 month ★b) no, if median duration of follow-up < 18 months | X  🞏 |
| 3. Adequacy of follow up of cohortsa) complete follow up: all subjects accounted for ★b) subjects lost to follow up unlikely to introduce bias: number lost <= 20%, ★ or description of those lost suggesting no different from those followedc) follow up rate < 80% (select an adequate %) and no description of those lostd) no statement | 🞏  X  🞏  🞏 |

NB Underlined text ‘customised’ for the intervention being reviewed

**Group:** Seddon **Study :** Culture confirmed multidrug resistant tuberculosis in children: clinical features, treatment and outcomes

| **Assessment of quality of a cohort study – Newcastle Ottawa Scale** |  |
| --- | --- |
| **Selection** (tick one box in each section) |  |
| 1. Representativeness of the study cohorta) truly representative of the average, 0-14 year old, MDR patient initiated on MDR-TB treatment (bacteriologically confirmed or clinically diagnosed) ★b) somewhat representative of the average, MDR patient ★c) selected group of patients, e.g. only certain socio-economic groups/areasd) no description of the derivation of the cohort | X  🞏  🞏  🞏 |
| 2. ~~Selection of the non intervention cohort~~~~a) drawn from the same community as the intervention cohort ★~~~~b) drawn from a different source~~~~c) no description of the derivation of the non intervention cohort~~ | 🞏  🞏  🞏 |
| 2. Ascertainment of interventiona) secure record (eg health care record) ★b) structured interview ★c) written self reportd) other / no description | X  🞏  🞏  🞏 |
| 4. Demonstration that outcome of interest was not present at start of studya) yes ★b) no | 🞏  X |
| Comparability (tick one or both boxes, as appropriate) |  |
| 1. Comparability of cohorts on the basis of the design or analysisa) study controls for age, HIV infection, ★b) study controls for any additional factors (bacteriological status, disease severity, nutritional status) ★ | X  X |
| Outcome (tick one box in each section) |  |
| 1. Assessment of outcomea) independent blind assessment ★b) record linkage ★c) self report **d) other / no description** | X  🞏  🞏  🞏 |
| 2. Was follow up long enough for outcomes to occura) yes, if median duration of follow-up >= 18 month ★b) no, if median duration of follow-up < 18 months | X  🞏 |
| 3. Adequacy of follow up of cohortsa) complete follow up: all subjects accounted for ★b) subjects lost to follow up unlikely to introduce bias: number lost <= 20%, ★ or description of those lost suggesting no different from those followedc) follow up rate < 80% (select an adequate %) and no description of those lostd) no statement | 🞏  X  🞏  🞏 |

NB Underlined text ‘customised’ for the intervention being reviewed

**Group:** Seddon **Study :** High treatment success in children treated for multidrug resistant tuberculosis: an observational cohort study

| **Assessment of quality of a cohort study – Newcastle Ottawa Scale** |  |
| --- | --- |
| **Selection** (tick one box in each section) |  |
| 1. Representativeness of the study cohorta) truly representative of the average, 0-14 year old, MDR patient initiated on MDR-TB treatment (bacteriologically confirmed or clinically diagnosed) ★b) somewhat representative of the average, MDR patient ★c) selected group of patients, e.g. only certain socio-economic groups/areasd) no description of the derivation of the cohort | X  🞏  🞏  🞏 |
| 2. ~~Selection of the non intervention cohort~~~~a) drawn from the same community as the intervention cohort ★~~~~b) drawn from a different source~~~~c) no description of the derivation of the non intervention cohort~~ | 🞏  🞏  🞏 |
| 2. Ascertainment of interventiona) secure record (eg health care record) ★b) structured interview ★c) written self reportd) other / no description | X  🞏  🞏  🞏 |
| 4. Demonstration that outcome of interest was not present at start of studya) yes ★b) no | 🞏  X |
| Comparability (tick one or both boxes, as appropriate) |  |
| 1. Comparability of cohorts on the basis of the design or analysisa) study controls for age, HIV infection, ★b) study controls for any additional factors (bacteriological status, disease severity, nutritional status) ★ | X  X |
| Outcome (tick one box in each section) |  |
| 1. Assessment of outcomea) independent blind assessment ★b) record linkage ★c) self report **d) other / no description** | X  🞏  🞏  🞏 |
| 2. Was follow up long enough for outcomes to occura) yes, if median duration of follow-up >= 18 month ★b) no, if median duration of follow-up < 18 months | X  🞏 |
| 3. Adequacy of follow up of cohortsa) complete follow up: all subjects accounted for ★b) subjects lost to follow up unlikely to introduce bias: number lost <= 20%, ★ or description of those lost suggesting no different from those followedc) follow up rate < 80% (select an adequate %) and no description of those lostd) no statement | 🞏  X  🞏  🞏 |

NB Underlined text ‘customised’ for the intervention being reviewed

**Group:** Swaminathan **Study :** Treating children for drug-resistant tuberculosis in Tajikistan with Group 5 medications

| **Assessment of quality of a cohort study – Newcastle Ottawa Scale** |  |
| --- | --- |
| **Selection** (tick one box in each section) |  |
| 1. Representativeness of the study cohorta) truly representative of the average, 0-14 year old, MDR patient initiated on MDR-TB treatment (bacteriologically confirmed or clinically diagnosed) ★b) somewhat representative of the average, MDR patient ★c) selected group of patients, e.g. only certain socio-economic groups/areasd) no description of the derivation of the cohort | X  🞏  🞏  🞏 |
| 2. ~~Selection of the non intervention cohort~~~~a) drawn from the same community as the intervention cohort ★~~~~b) drawn from a different source~~~~c) no description of the derivation of the non intervention cohort~~ | 🞏  🞏  🞏 |
| 2. Ascertainment of interventiona) secure record (eg health care record) ★b) structured interview ★c) written self reportd) other / no description | X  🞏  🞏  🞏 |
| 4. Demonstration that outcome of interest was not present at start of studya) yes ★b) no | 🞏  X |
| Comparability (tick one or both boxes, as appropriate) |  |
| 1. Comparability of cohorts on the basis of the design or analysisa) study controls for age, HIV infection, ★b) study controls for any additional factors (bacteriological status, disease severity, nutritional status) ★ | X  🞏 |
| Outcome (tick one box in each section) |  |
| 1. Assessment of outcomea) independent blind assessment ★b) record linkage ★c) self report **d) other / no description** | X  🞏  🞏  🞏 |
| 2. Was follow up long enough for outcomes to occura) yes, if median duration of follow-up >= 18 month ★b) no, if median duration of follow-up < 18 months | 🞏  🞏 |
| 3. Adequacy of follow up of cohortsa) complete follow up: all subjects accounted for ★b) subjects lost to follow up unlikely to introduce bias: number lost <= 20%, ★ or description of those lost suggesting no different from those followedc) follow up rate < 80% (select an adequate %) and no description of those lostd) no statement | 🞏  🞏  X  🞏 |

NB Underlined text ‘customised’ for the intervention being reviewed

**Group:** Williams **Study :** Multidrug-resistant tuberculosis in UK children: presentation, management and outcome

| **Assessment of quality of a cohort study – Newcastle Ottawa Scale** |  |
| --- | --- |
| **Selection** (tick one box in each section) |  |
| 1. Representativeness of the study cohorta) truly representative of the average, 0-14 year old, MDR patient initiated on MDR-TB treatment (bacteriologically confirmed or clinically diagnosed) ★b) somewhat representative of the average, MDR patient ★c) selected group of patients, e.g. only certain socio-economic groups/areasd) no description of the derivation of the cohort | X  🞏  🞏  🞏 |
| 2. ~~Selection of the non intervention cohort~~~~a) drawn from the same community as the intervention cohort ★~~~~b) drawn from a different source~~~~c) no description of the derivation of the non intervention cohort~~ | 🞏  🞏  🞏 |
| 2. Ascertainment of interventiona) secure record (eg health care record) ★b) structured interview ★c) written self reportd) other / no description | X  🞏  🞏  🞏 |
| 4. Demonstration that outcome of interest was not present at start of studya) yes ★b) no | 🞏  X |
| Comparability (tick one or both boxes, as appropriate) |  |
| 1. Comparability of cohorts on the basis of the design or analysisa) study controls for age, HIV infection, ★b) study controls for any additional factors (bacteriological status, disease severity, nutritional status) ★ | X  🞏 |
| Outcome (tick one box in each section) |  |
| 1. Assessment of outcomea) independent blind assessment ★b) record linkage ★c) self report **d) other / no description** | X  🞏  🞏  🞏 |
| 2. Was follow up long enough for outcomes to occura) yes, if median duration of follow-up >= 18 month ★b) no, if median duration of follow-up < 18 months | 🞏  🞏 |
| 3. Adequacy of follow up of cohortsa) complete follow up: all subjects accounted for ★b) subjects lost to follow up unlikely to introduce bias: number lost <= 20%, ★ or description of those lost suggesting no different from those followedc) follow up rate < 80% (select an adequate %) and no description of those lostd) no statement | 🞏  🞏  X  🞏 |

NB Underlined text ‘customised’ for the intervention being reviewed
