# Supplementary material for: Treatment and outcomes in children with multidrug-resistant tuberculosis: A systematic review and individual patient data meta-analysis
Source: PLoS Med. 2018 Jul 11;15(7):e1002591. doi: 10.1371/journal.pmed.1002591 (PMC6040687; doi:10.1371/journal.pmed.1002591)
Supplement: S3 Table — (DOCX) [file pmed.1002591.s003.docx]

**S3 Table. Overview of included studies and cohorts**

| **Site/author** | **Country** | **Reference**  **Number** | **Previous IPD inclusion** | **Number of subjects** | **Publication status** | **Reference** | **Study design** | **Study population** | **Data Request Contact Organization** |
| --- | --- | --- | --- | --- | --- | --- | --- | --- | --- |
| Achar | Uzbekistan | N/A | No | 20 | Unpublished | N/A | Retrospective cohort | All consecutively presenting bacteriologically confirmed and clinically diagnosed patients | Médecins Sans Frontières (MSF) Field Research Policy (<http://hdl.handle.net/10144/306501>); and data sharing repository ([data.sharing@msf.org](mailto:data.sharing@msf.org)) Amsterdam, Holland. Contact: Dr Sidney Wong, Medical Director, MSF Operational Centre Amsterdam, Holland ([Sidney.Wong@amsterdam.msf.org](mailto:Sidney.Wong@amsterdam.msf.org)) |
| Amanullah | Pakistan | N/A | No | 29 | Unpublished | N/A | Retrospective cohort | All consecutively presenting bacteriologically confirmed patients | Indus Hospital Research Center/ Interactive Research and Development. Contact:  http://ird.global/irb/ |
| Chan | Taiwan | N/A | No | 4 | Unpublished | N/A | Retrospective cohort | All consecutively presenting bacteriologically confirmed patients | Institutional Review Board; Centers for Disease Control; Ministry of Health and Welfare; Taiwan. Contact:  [ihrfocalpoint@cdc.gov.tw](http://www.cdc.gov.tw/mailto:ihrfocalpoint@cdc.gov.tw) |
| Chan | USA | 12 | Menzies IPD 2012 | 3 | **Published** | [Chan ED](http://www.ncbi.nlm.nih.gov/pubmed/?term=Chan%20ED%5BAuthor%5D&cauthor=true&cauthor_uid=14742301), [Laurel V](http://www.ncbi.nlm.nih.gov/pubmed/?term=Laurel%20V%5BAuthor%5D&cauthor=true&cauthor_uid=14742301), [Strand MJ](http://www.ncbi.nlm.nih.gov/pubmed/?term=Strand%20MJ%5BAuthor%5D&cauthor=true&cauthor_uid=14742301), [Chan JF](http://www.ncbi.nlm.nih.gov/pubmed/?term=Chan%20JF%5BAuthor%5D&cauthor=true&cauthor_uid=14742301), [Huynh ML](http://www.ncbi.nlm.nih.gov/pubmed/?term=Huynh%20ML%5BAuthor%5D&cauthor=true&cauthor_uid=14742301), [Goble M](http://www.ncbi.nlm.nih.gov/pubmed/?term=Goble%20M%5BAuthor%5D&cauthor=true&cauthor_uid=14742301), et al. Treatment and outcome analysis of 205 patients with multidrug-resistant tuberculosis. Am J Respir Crit Care Med 2004 May 15;169(10):1103-9. | Retrospective cohort | All consecutively presenting bacteriologically confirmed patients | National Jewish Health Institutional Review Board, Denver, USA. Contact: https://www.nationaljewish.org/research-science/support/compliance/irb |
| Chiotan | Romania | N/A | No | 17 | Unpublished | N/A | Retrospective cohort | All consecutively presenting bacteriologically confirmed patients | Marius Nasta Pneumology Institute; Bucharest, Romania. Contact: secretariat@marius-nasta.ro |
| Datta | India | 19 | No | 3 | **Published** | [Datta BS](http://www.ncbi.nlm.nih.gov/pubmed/?term=Datta%20BS%5BAuthor%5D&cauthor=true&cauthor_uid=20130374), [Hassan G](http://www.ncbi.nlm.nih.gov/pubmed/?term=Hassan%20G%5BAuthor%5D&cauthor=true&cauthor_uid=20130374), [Kadri SM](http://www.ncbi.nlm.nih.gov/pubmed/?term=Kadri%20SM%5BAuthor%5D&cauthor=true&cauthor_uid=20130374), [Qureshi W](http://www.ncbi.nlm.nih.gov/pubmed/?term=Qureshi%20W%5BAuthor%5D&cauthor=true&cauthor_uid=20130374), [Kamili MA](http://www.ncbi.nlm.nih.gov/pubmed/?term=Kamili%20MA%5BAuthor%5D&cauthor=true&cauthor_uid=20130374), [Singh H](http://www.ncbi.nlm.nih.gov/pubmed/?term=Singh%20H%5BAuthor%5D&cauthor=true&cauthor_uid=20130374), et al. Multidrug-resistant and extensively drug resistant tuberculosis in Kashmir, India. [J Infect Dev Ctries.](http://www.ncbi.nlm.nih.gov/pubmed/?term=Multidrug-resistant+and+extensively+drug+resistant+tuberculosis+in+Kashmir%2C+India) 2009 Nov 21;4(1):19-23.. | Prospective cohort | All consecutively presenting bacteriologically confirmed pulmonary TB patients | Government Medical College Associated Chest Diseases Hospital, Srinagar, Kashmir, India. Contact: http://www.gmcs.edu.in/Administrative_Block |
| Drobac | Peru | 13 | No | 36 | **Published** | [Drobac PC](http://www.ncbi.nlm.nih.gov/pubmed/?term=Drobac%20PC%5BAuthor%5D&cauthor=true&cauthor_uid=16740844), [Mukherjee JS](http://www.ncbi.nlm.nih.gov/pubmed/?term=Mukherjee%20JS%5BAuthor%5D&cauthor=true&cauthor_uid=16740844), [Joseph JK](http://www.ncbi.nlm.nih.gov/pubmed/?term=Joseph%20JK%5BAuthor%5D&cauthor=true&cauthor_uid=16740844), [Mitnick C](http://www.ncbi.nlm.nih.gov/pubmed/?term=Mitnick%20C%5BAuthor%5D&cauthor=true&cauthor_uid=16740844), [Furin JJ](http://www.ncbi.nlm.nih.gov/pubmed/?term=Furin%20JJ%5BAuthor%5D&cauthor=true&cauthor_uid=16740844), [del Castillo H](http://www.ncbi.nlm.nih.gov/pubmed/?term=del%20Castillo%20H%5BAuthor%5D&cauthor=true&cauthor_uid=16740844), et al. Community-based therapy for children with multidrug-resistant tuberculosis. [Pediatrics.](http://www.ncbi.nlm.nih.gov/pubmed/16740844) 2006 Jun;117(6):2022-9. | Retrospective cohort | All consecutively presenting bacteriologically confirmed and clinically diagnosed patients | Harvard Medical School, Office of Human Research Administration, Boston USA. Contact: <https://www.hsph.harvard.edu/ohra/about-us/>  [Leslie Howes](https://www.hsph.harvard.edu/ohra/contacts-locations/staff-contact-information/leslie-howes/), MPH, CIP Managing Director Phone: +1 617-432-2153 Email: [lhowes@hsph.harvard.edu](mailto:lhowes@hsph.harvard.edu) |
| Fairlie | South Africa | 14 | No | 10 | **Published** | [Fairlie L](http://www.ncbi.nlm.nih.gov/pubmed/?term=Fairlie%20L%5BAuthor%5D&cauthor=true&cauthor_uid=21269475), [Beylis NC](http://www.ncbi.nlm.nih.gov/pubmed/?term=Beylis%20NC%5BAuthor%5D&cauthor=true&cauthor_uid=21269475), [Reubenson G](http://www.ncbi.nlm.nih.gov/pubmed/?term=Reubenson%20G%5BAuthor%5D&cauthor=true&cauthor_uid=21269475), [Moore DP](http://www.ncbi.nlm.nih.gov/pubmed/?term=Moore%20DP%5BAuthor%5D&cauthor=true&cauthor_uid=21269475), [Madhi SA](http://www.ncbi.nlm.nih.gov/pubmed/?term=Madhi%20SA%5BAuthor%5D&cauthor=true&cauthor_uid=21269475). High prevalence of childhood multi-drug resistant tuberculosis in Johannesburg, South Africa: a cross sectional study. [BMC Infect Dis.](http://www.ncbi.nlm.nih.gov/pubmed/?term=High+prevalence+of+childhood+multi-drug+resistant+tuberculosis+in+Johannesburg%2C+South+Africa%3A+a+cross+sectional+study) 2011 Jan 26;11:28. | Retrospective cohort | All consecutively presenting bacteriologically confirmed patients | University of the Witwatersrand, Human Research Ethics Committee (Medical), Johannesburg, South Africa. Contact: https://www.wits.ac.za/ethics/human-research-ethics-committee-medical/ |
| Geerligs | Netherlands | 27 | Menzies IPD 2014 | 2 | **Published** | [Geerligs WA](http://www.ncbi.nlm.nih.gov/pubmed/?term=Geerligs%20WA%5BAuthor%5D&cauthor=true&cauthor_uid=10949328), [Van Altena R](http://www.ncbi.nlm.nih.gov/pubmed/?term=Van%20Altena%20R%5BAuthor%5D&cauthor=true&cauthor_uid=10949328), [De Lange WCM](http://www.ncbi.nlm.nih.gov/pubmed/?term=De%20Lange%20WCM%5BAuthor%5D&cauthor=true&cauthor_uid=10949328), [Van Soolingen D](http://www.ncbi.nlm.nih.gov/pubmed/?term=Van%20Soolingen%20D%5BAuthor%5D&cauthor=true&cauthor_uid=10949328), [Van Der Werf TS](http://www.ncbi.nlm.nih.gov/pubmed/?term=Van%20Der%20Werf%20TS%5BAuthor%5D&cauthor=true&cauthor_uid=10949328). Multidrug-resistant tuberculosis: long-term treatment outcome in the Netherlands. [Int J Tuberc Lung Dis.](http://www.ncbi.nlm.nih.gov/pubmed/10949328) 2000 Aug; 4(8): 758-64 | Retrospective cohort | All consecutively presenting bacteriologically confirmed patients | Groningen University Hospital, Research Office UMCG, Groningen, the Netherlands. Contact: https://www.umcg.nl/EN/Research/Researchers/Facilities/ResearchOffice/Paginas/default.aspx |
| Gegia | Georgia | 15 | No | 55 | **Published** | [Gegia M](http://www.ncbi.nlm.nih.gov/pubmed/?term=Gegia%20M%5BAuthor%5D&cauthor=true&cauthor_uid=23575328), [Jenkins HE](http://www.ncbi.nlm.nih.gov/pubmed/?term=Jenkins%20HE%5BAuthor%5D&cauthor=true&cauthor_uid=23575328), [Kalandadze I](http://www.ncbi.nlm.nih.gov/pubmed/?term=Kalandadze%20I%5BAuthor%5D&cauthor=true&cauthor_uid=23575328), [Furin J](http://www.ncbi.nlm.nih.gov/pubmed/?term=Furin%20J%5BAuthor%5D&cauthor=true&cauthor_uid=23575328). Outcomes of children treated for tuberculosis with second-line medications in Georgia, 2009-2011. [Int J Tuberc Lung Dis.](http://www.ncbi.nlm.nih.gov/pubmed/?term=Outcomes+of+children+treated+for+tuberculosis+with+second-line+medications+in+Georgia%2C+2009-2011) 2013 May;17(5):624-9. | Retrospective cohort | All consecutively presenting bacteriologically confirmed and clinically diagnosed patients | Case Western Reserve University, Institutional Review Board, Cleveland, USA. Contact: http://case.edu/research/faculty-staff/compliance/irb/ |
| Granich | USA | 16 | Menzies IPD 2013 | 3 | **Published** | Granich RM, [Oh P](http://www.ncbi.nlm.nih.gov/pubmed/?term=Oh%20P%5BAuthor%5D&cauthor=true&cauthor_uid=18616396), Lewis B, Porco TC, Flood J. Multidrug resistance among persons with tuberculosis in California, 1994-2003. [JAMA.](http://www.ncbi.nlm.nih.gov/pubmed/?term=Extensively+drug-resistant+tuberculosis+in+California%2C+1993%E2%80%932006) 2005; 293: 2732-2739. | Retrospective cohort | All consecutively presenting bacteriologically confirmed patients | State of California; Health and Human Services Agency; Committee for the Protection of Human Subjects. Contact: +1 (916) 326-3660 |
| Hicks | South Africa | 17 | No | 82 | **Published** | [Hicks RM](http://www.ncbi.nlm.nih.gov/pubmed/?term=Hicks%20RM%5BAuthor%5D&cauthor=true&cauthor_uid=25189555), [Padayatchi N](http://www.ncbi.nlm.nih.gov/pubmed/?term=Padayatchi%20N%5BAuthor%5D&cauthor=true&cauthor_uid=25189555), [Shah NS](http://www.ncbi.nlm.nih.gov/pubmed/?term=Shah%20NS%5BAuthor%5D&cauthor=true&cauthor_uid=25189555), [Wolf A](http://www.ncbi.nlm.nih.gov/pubmed/?term=Wolf%20A%5BAuthor%5D&cauthor=true&cauthor_uid=25189555), [Werner L](http://www.ncbi.nlm.nih.gov/pubmed/?term=Werner%20L%5BAuthor%5D&cauthor=true&cauthor_uid=25189555), [Sunkari VB](http://www.ncbi.nlm.nih.gov/pubmed/?term=Sunkari%20VB%5BAuthor%5D&cauthor=true&cauthor_uid=25189555), [et al](http://www.ncbi.nlm.nih.gov/pubmed/?term=O%27Donnell%20MR%5BAuthor%5D&cauthor=true&cauthor_uid=25189555). Malnutrition associated with unfavorable outcome and death among South African MDR-TB and HIV co-infected children. [Int J Tuberc Lung Dis.](http://www.ncbi.nlm.nih.gov/pubmed/?term=Malnutrition+associated+with+unfavourable+outcomes+and+death+among+South+African+MDR-TB+and+HIV+co-infected+children) 2014 Sep;18(9):1074-83. | Retrospective cohort | All consecutively presenting bacteriologically confirmed patients | Albert Einstein College of Medicine, Institutional Review Board, New York, USA. Contact: https://www.einstein.yu.edu/administration/institutional-review-board/ |
| Isaakidis | India | 18 | No | 8 | **Published** | [Isaakidis P](http://www.ncbi.nlm.nih.gov/pubmed/?term=Isaakidis%20P%5BAuthor%5D&cauthor=true&cauthor_uid=23894358), [Paryani R](http://www.ncbi.nlm.nih.gov/pubmed/?term=Paryani%20R%5BAuthor%5D&cauthor=true&cauthor_uid=23894358), [Khan S](http://www.ncbi.nlm.nih.gov/pubmed/?term=Khan%20S%5BAuthor%5D&cauthor=true&cauthor_uid=23894358), [Mansoor H](http://www.ncbi.nlm.nih.gov/pubmed/?term=Mansoor%20H%5BAuthor%5D&cauthor=true&cauthor_uid=23894358), [Manglani M](http://www.ncbi.nlm.nih.gov/pubmed/?term=Manglani%20M%5BAuthor%5D&cauthor=true&cauthor_uid=23894358), [Valiyakath A](http://www.ncbi.nlm.nih.gov/pubmed/?term=Valiyakath%20A%5BAuthor%5D&cauthor=true&cauthor_uid=23894358), et al. Poor outcomes in a cohort of HIV-infected adolescents undergoing treatment for multidrug-resistant tuberculosis in Mumbai, India. [PLoS One.](http://www.ncbi.nlm.nih.gov/pubmed/?term=Poor+outcomes+in+a+cohort+of+HIV-infected+adolescents+undergoing+treatment+for+multidrug-resistant+tuberculosis+in+Mumbai%2C+India.) 2013 Jul 19; 8(7):e68869.. | Retrospective cohort | All consecutively presenting bacteriologically confirmed patients | Médecins Sans Frontières (MSF) Field Research Policy (<http://hdl.handle.net/10144/306501>); and data sharing repository ([data.sharing@msf.org](mailto:data.sharing@msf.org)), Brussels, Belgium. Contact: Dr Sebastian Spencer, Medical Director, MSF-Operational Center Brussels, Brussels, Belgium  ([Sebastian.Spencer@brussels.msf.org](mailto:Sebastian.Spencer@brussels.msf.org)) |
| Kim /Shim | Korea | 26 | Menzies IPD 2014 | 2 | **Published** | [Kim DH](http://www.ncbi.nlm.nih.gov/pubmed/?term=Kim%20DH%5BAuthor%5D&cauthor=true&cauthor_uid=18703792), [Kim HJ](http://www.ncbi.nlm.nih.gov/pubmed/?term=Kim%20HJ%5BAuthor%5D&cauthor=true&cauthor_uid=18703792), [Park SK](http://www.ncbi.nlm.nih.gov/pubmed/?term=Park%20SK%5BAuthor%5D&cauthor=true&cauthor_uid=18703792), [Kong SJ](http://www.ncbi.nlm.nih.gov/pubmed/?term=Kong%20SJ%5BAuthor%5D&cauthor=true&cauthor_uid=18703792), [Kim YS](http://www.ncbi.nlm.nih.gov/pubmed/?term=Kim%20YS%5BAuthor%5D&cauthor=true&cauthor_uid=18703792), [Kim TH](http://www.ncbi.nlm.nih.gov/pubmed/?term=Kim%20TH%5BAuthor%5D&cauthor=true&cauthor_uid=18703792), et al. Treatment outcomes and long-term survival in patients with extensively drug-resistant tuberculosis. [Am J Respir Crit Care Med.](http://www.ncbi.nlm.nih.gov/pubmed/18703792) 2008 Nov 15;178(10):1075-82. | Retrospective cohort | All consecutively presenting bacteriologically confirmed patients | Asan Medical Center, Seoul, South Korea.  Contact: http://eirb.amc.seoul.kr |
| Kim/Yim | Korea | 29 | Menzies IPD 2014 | 1 | **Published** | [Kim HR](http://www.ncbi.nlm.nih.gov/pubmed/?term=Kim%20HR%5BAuthor%5D&cauthor=true&cauthor_uid=17968823), [Hwang SS](http://www.ncbi.nlm.nih.gov/pubmed/?term=Hwang%20SS%5BAuthor%5D&cauthor=true&cauthor_uid=17968823), [Kim HJ](http://www.ncbi.nlm.nih.gov/pubmed/?term=Kim%20HJ%5BAuthor%5D&cauthor=true&cauthor_uid=17968823), [Lee SM](http://www.ncbi.nlm.nih.gov/pubmed/?term=Lee%20SM%5BAuthor%5D&cauthor=true&cauthor_uid=17968823), [Yoo CG](http://www.ncbi.nlm.nih.gov/pubmed/?term=Yoo%20CG%5BAuthor%5D&cauthor=true&cauthor_uid=17968823), [Kim YW](http://www.ncbi.nlm.nih.gov/pubmed/?term=Kim%20YW%5BAuthor%5D&cauthor=true&cauthor_uid=17968823), [Han SK](http://www.ncbi.nlm.nih.gov/pubmed/?term=Han%20SK%5BAuthor%5D&cauthor=true&cauthor_uid=17968823), et al. Impact of extensive drug resistance on treatment outcomes in non-HIV-infected patients with multidrug-resistant tuberculosis. [Clin Infect Dis.](http://www.ncbi.nlm.nih.gov/pubmed/?term=Impact+of+extensive+drug+resistance+on+treatment+outcomes+in+non-HIV-infected+patients+with+multidrug-resistant+tuberculosis) 2007 Nov 15;45(10):1290-5. | Retrospective cohort | All consecutively presenting HIV-negative bacteriologically confirmed XDR-TB patients | Seoul National University Hospital, Center for Human Research Protection Seoul, South Korea. Contact: http://m.snuh.org/english/snuh/snuh03/sub02/index2.jsp#no7 |
| Kuksa/Ozere | Latvia | 22 | No | 53 | **Partly published** | [Kuksa L](http://www.ncbi.nlm.nih.gov/pubmed/?term=Kuksa%20L%5BAuthor%5D&cauthor=true&cauthor_uid=26393098), [Riekstina V](http://www.ncbi.nlm.nih.gov/pubmed/?term=Riekstina%20V%5BAuthor%5D&cauthor=true&cauthor_uid=26393098), [Leimane V](http://www.ncbi.nlm.nih.gov/pubmed/?term=Leimane%20V%5BAuthor%5D&cauthor=true&cauthor_uid=26393098), [Ozere I](http://www.ncbi.nlm.nih.gov/pubmed/?term=Ozere%20I%5BAuthor%5D&cauthor=true&cauthor_uid=26393098), [Skenders G](http://www.ncbi.nlm.nih.gov/pubmed/?term=Skenders%20G%5BAuthor%5D&cauthor=true&cauthor_uid=26393098), [Van den Bergh R](http://www.ncbi.nlm.nih.gov/pubmed/?term=Van%20den%20Bergh%20R%5BAuthor%5D&cauthor=true&cauthor_uid=26393098), et al. Multi- and extensively drug-resistant tuberculosis in Latvia: trends, characteristics and treatment outcomes. [Public Health Action.](http://www.ncbi.nlm.nih.gov/pubmed/?term=Multi-+and+extensively+drug-resistant+tuberculosis+in+Latvia%3A+trends%2C+characteristics+and+treatment+outcomes.) 2014 Oct 21;4(Suppl 2):S47-53. | Retrospective cohort | All consecutively presenting bacteriologically confirmed and clinically diagnosed patients | Riga Stradins University, Research Department, Riga, Latvia. Contact: https://www.rsu.lv/en/research-department |
| Mariandyshev | Russia | N/A | No | 38 | **Published** | [Smirnova PA](https://www.ncbi.nlm.nih.gov/pubmed/?term=Smirnova%20PA%5BAuthor%5D&cauthor=true&cauthor_uid=27587542), [Turkova A](https://www.ncbi.nlm.nih.gov/pubmed/?term=Turkova%20A%5BAuthor%5D&cauthor=true&cauthor_uid=27587542), [Nikishova EI](https://www.ncbi.nlm.nih.gov/pubmed/?term=Nikishova%20EI%5BAuthor%5D&cauthor=true&cauthor_uid=27587542), [Seddon JA](https://www.ncbi.nlm.nih.gov/pubmed/?term=Seddon%20JA%5BAuthor%5D&cauthor=true&cauthor_uid=27587542), [Chappell E](https://www.ncbi.nlm.nih.gov/pubmed/?term=Chappell%20E%5BAuthor%5D&cauthor=true&cauthor_uid=27587542), [Zolotaya OA](https://www.ncbi.nlm.nih.gov/pubmed/?term=Zolotaya%20OA%5BAuthor%5D&cauthor=true&cauthor_uid=27587542), [Mironuk OM](https://www.ncbi.nlm.nih.gov/pubmed/?term=Mironuk%20OM%5BAuthor%5D&cauthor=true&cauthor_uid=27587542), [Maryandyshev AO](https://www.ncbi.nlm.nih.gov/pubmed/?term=Maryandyshev%20AO%5BAuthor%5D&cauthor=true&cauthor_uid=27587542). Multidrug-resistant tuberculosis in children in northwest Russia: an observational cohort study. [Eur Respir J.](https://www.ncbi.nlm.nih.gov/pubmed/27587542) 2016 Nov;48(5):1496-1499 | Retrospective cohort | All consecutively presenting bacteriologically confirmed and clinically diagnosed patients | Northern State Medical University, Arkhangelsk, Russian Federation. Contact: http://www.nsmu.ru/eng/ |
| Mendez-Echevarria | Spain | 20 | No | 8 | **Published** | [Méndez Echevarría A](http://www.ncbi.nlm.nih.gov/pubmed/?term=M%C3%A9ndez%20Echevarr%C3%ADa%20A%5BAuthor%5D&cauthor=true&cauthor_uid=17785156), [Baquero Artigao F](http://www.ncbi.nlm.nih.gov/pubmed/?term=Baquero%20Artigao%20F%5BAuthor%5D&cauthor=true&cauthor_uid=17785156), [García Miguel MJ](http://www.ncbi.nlm.nih.gov/pubmed/?term=Garc%C3%ADa%20Miguel%20MJ%5BAuthor%5D&cauthor=true&cauthor_uid=17785156), [Rojo Conejo P](http://www.ncbi.nlm.nih.gov/pubmed/?term=Rojo%20Conejo%20P%5BAuthor%5D&cauthor=true&cauthor_uid=17785156), [Ballesteros Díez Y](http://www.ncbi.nlm.nih.gov/pubmed/?term=Ballesteros%20D%C3%ADez%20Y%5BAuthor%5D&cauthor=true&cauthor_uid=17785156), [Rubio Gribble B](http://www.ncbi.nlm.nih.gov/pubmed/?term=Rubio%20Gribble%20B%5BAuthor%5D&cauthor=true&cauthor_uid=17785156), et al. Multidrug-resistant tuberculosis in the pediatric age group. [An Pediatr (Barc).](http://www.ncbi.nlm.nih.gov/pubmed/17785156) 2007 Sep;67(3):206-11. | Retrospective cohort | All consecutively presenting bacteriologically confirmed and clinically diagnosed patients | La Paz Universitary Hospital.  Institute for Health Research IdiPAZ. Madrid, Spain.  Contact:  Phone/Fax: +34 91 7277479 |
| Moore | South Africa | 21 | No | 339 | **Published** | [Moore BK](http://www.ncbi.nlm.nih.gov/pubmed/?term=Moore%20BK%5BAuthor%5D&cauthor=true&cauthor_uid=25946356), [Anyalechi E](http://www.ncbi.nlm.nih.gov/pubmed/?term=Anyalechi%20E%5BAuthor%5D&cauthor=true&cauthor_uid=25946356), [van der Walt M](http://www.ncbi.nlm.nih.gov/pubmed/?term=van%20der%20Walt%20M%5BAuthor%5D&cauthor=true&cauthor_uid=25946356), [Smith S](http://www.ncbi.nlm.nih.gov/pubmed/?term=Smith%20S%5BAuthor%5D&cauthor=true&cauthor_uid=25946356), [Erasmus L](http://www.ncbi.nlm.nih.gov/pubmed/?term=Erasmus%20L%5BAuthor%5D&cauthor=true&cauthor_uid=25946356), [Lancaster J](http://www.ncbi.nlm.nih.gov/pubmed/?term=Lancaster%20J%5BAuthor%5D&cauthor=true&cauthor_uid=25946356), et al. Epidemiology of drug-resistant tuberculosis among children and adolescents in South Africa, 2005-2010. [Int J Tuberc Lung Dis.](http://www.ncbi.nlm.nih.gov/pubmed/?term=Epidemiology+of+drug-resistant+tuberculosis+among+children+and+adolescents+in+South+Africa%2C+2005%E2%80%932010) 2015 Jun;19(6):663-9. | Retrospective cohort | All consecutively presenting bacteriologically confirmed and clinically diagnosed patients | Centers for Disease Control and Prevention (CDC), Institutional Review Board, Atlanta, USA. Contact: https://www.cdc.gov/od/science/integrity/hrpo/irbs.htm |
| Munsiff | USA | 31 | Menzies IPD 2012 | 11 | **Published** | Munsiff SS, Ahuja SD, Li J, Driver CR. Public-private collaboration for multidrug-resistant tuberculosis control in New York City. Int J Tuberc Lung Dis. 2006; 10: 639–648. | Retrospective cohort | All consecutively presenting bacteriologically confirmed patients | New York City Department of Health and Mental Hygiene, Institutional Human Subjects Review Board of the NYC Health Department, New York City, USA. Contact: https://www1.nyc.gov/site/doh/about/about-doh/institutional-review-board.page |
| Padayatchi | South Africa | N/A | No | 4 | Unpublished | N/A | Retrospective cohort | All consecutively presenting bacteriologically confirmed patients | University of KwaZulu-Natal; Biomedical Research Ethics Administration; KwaZulu-Natal, South Africa; Contact: BREC@ukzn.ac.za |
| Rybak | Ukraine | N/A | No | 6 | Unpublished | N/A | Retrospective cohort | All consecutively presenting bacteriologically confirmed and clinically diagnosed patients | Lifespan/ The Miriam Hospital Institutional Review Board  Providence, RI USA  Contact:  <https://www.lifespan.org/office-research-administration/institutional-review-board-irb> |
| Santiago-Garcia | Spain | 23 | No | 10 | **Partly published** | [Santiago B](http://www.ncbi.nlm.nih.gov/pubmed/?term=Santiago%20B%5BAuthor%5D&cauthor=true&cauthor_uid=24622395), [Baquero-Artigao F](http://www.ncbi.nlm.nih.gov/pubmed/?term=Baquero-Artigao%20F%5BAuthor%5D&cauthor=true&cauthor_uid=24622395), [Mejías A](http://www.ncbi.nlm.nih.gov/pubmed/?term=Mej%C3%ADas%20A%5BAuthor%5D&cauthor=true&cauthor_uid=24622395), [Blázquez D](http://www.ncbi.nlm.nih.gov/pubmed/?term=Bl%C3%A1zquez%20D%5BAuthor%5D&cauthor=true&cauthor_uid=24622395), [Jiménez MS](http://www.ncbi.nlm.nih.gov/pubmed/?term=Jim%C3%A9nez%20MS%5BAuthor%5D&cauthor=true&cauthor_uid=24622395), [Mellado-Peña MJ](http://www.ncbi.nlm.nih.gov/pubmed/?term=Mellado-Pe%C3%B1a%20MJ%5BAuthor%5D&cauthor=true&cauthor_uid=24622395); [EREMITA Study Group](http://www.ncbi.nlm.nih.gov/pubmed/?term=EREMITA%20Study%20Group%5BCorporate%20Author%5D). Pediatric drug-resistant tuberculosis in Madrid: family matters. [Pediatr Infect Dis J.](http://www.ncbi.nlm.nih.gov/pubmed/?term=Pediatric+drug-resistant+tuberculosis+in+Madrid%3A+family+matters) 2014 Apr;33(4):345-50. | Retrospective cohort | All consecutively presenting bacteriologically confirmed and clinically diagnosed patients | Secretaría Técnica Comité de Ética de la Investigación con Medicamentos.  Fundación para la Investigación Biomédica  Hospital Gregorio Marañón  Pabellón de Gobierno, 1ª planta  C/ Dr. Esquerdo, 46  28007 Madrid, Spain.  tlf: [+34 91 586 7007](tel:%2B34%2091%20586%207007)  e-mail: [ceim.hgugm@salud.madrid.org](mailto:ceim.hgugm@salud.madrid.org)  http://www.iisgm.com/en/organizacion/comisiones/comite-de-etica-de-la-investigacion-con-medicamentos-ceim~~/~~ |
| Seddon (1) | South Africa | 24 | No | 88 | **Published** | [Seddon JA](http://www.ncbi.nlm.nih.gov/pubmed/?term=Seddon%20JA%5BAuthor%5D&cauthor=true&cauthor_uid=22052896), [Hesseling AC](http://www.ncbi.nlm.nih.gov/pubmed/?term=Hesseling%20AC%5BAuthor%5D&cauthor=true&cauthor_uid=22052896), [Willemse M](http://www.ncbi.nlm.nih.gov/pubmed/?term=Willemse%20M%5BAuthor%5D&cauthor=true&cauthor_uid=22052896), [Donald PR](http://www.ncbi.nlm.nih.gov/pubmed/?term=Donald%20PR%5BAuthor%5D&cauthor=true&cauthor_uid=22052896), [Schaaf HS](http://www.ncbi.nlm.nih.gov/pubmed/?term=Schaaf%20HS%5BAuthor%5D&cauthor=true&cauthor_uid=22052896). Culture-confirmed multidrug-resistant tuberculosis in children: clinical features, treatment, and outcome. [Clin Infect Dis.](http://www.ncbi.nlm.nih.gov/pubmed/22052896) 2012 Jan 15;54(2):157-66. | Retrospective cohort | All consecutively presenting bacteriologically confirmed patients | Stellenbosch University; Health Research Ethics Committee; Cape Town, South Africa. Contact: 27 (0) 21-938-9657 |
| Seddon (2) | South Africa | 5 | No | 131 | **Published** | Seddon JA, Hesseling AC, Godfrey-Faussett P, Schaaf HS. [High treatment success in children treated for multidrug-resistant tuberculosis: an observational cohort study.](https://www.ncbi.nlm.nih.gov/pubmed/24064441) Thorax. 2014 May;69(5):458-64. | Prospective cohort | All consecutively presenting bacteriologically confirmed and clinically diagnosed patients | Stellenbosch University; Health Research Ethics Committee; Cape Town, South Africa. Contact: 27 (0) 21-938-9657 |
| Sharma | India | N/A | No | 8 | Unpublished | N/A | Retrospective cohort | All consecutively presenting bacteriologically confirmed patients | National Institute of Tuberculosis and Respiratory Diseases; Ethical Committee of National Institute; New Delhi, India. Contact: [www.lrsitbrd.nic.in](http://www.lrsitbrd.nic.in) |
| Skrahina | Belarus | N/A | No | 5 | Unpublished | N/A | Retrospective cohort | All consecutively presenting bacteriologically confirmed and clinically diagnosed patients | The Republican Research and Practical Centre for Pulmonology and TB, Minsk, Belarus; Contact:  Dolginovski tract 157, 220053, Minsk, Belarus  Tel: +375 17 2898352  Fax: +375 17 2898950  Dzklm99@yahoo.com  [niipulm@tut.by](mailto:niipulm@tut.by) |
| Swaminathan | Tajikistan | 30 | No | 27 | **Partly Published** | [Swaminathan A](https://www.ncbi.nlm.nih.gov/pubmed/?term=Swaminathan%20A%5BAuthor%5D&cauthor=true&cauthor_uid=26970156), [du Cros P](https://www.ncbi.nlm.nih.gov/pubmed/?term=du%20Cros%20P%5BAuthor%5D&cauthor=true&cauthor_uid=26970156), [Seddon JA](https://www.ncbi.nlm.nih.gov/pubmed/?term=Seddon%20JA%5BAuthor%5D&cauthor=true&cauthor_uid=26970156), [Quinnell S](https://www.ncbi.nlm.nih.gov/pubmed/?term=Quinnell%20S%5BAuthor%5D&cauthor=true&cauthor_uid=26970156), [Bobokhojaev OI](https://www.ncbi.nlm.nih.gov/pubmed/?term=Bobokhojaev%20OI%5BAuthor%5D&cauthor=true&cauthor_uid=26970156), [Dusmatova Z](https://www.ncbi.nlm.nih.gov/pubmed/?term=Dusmatova%20Z%5BAuthor%5D&cauthor=true&cauthor_uid=26970156), et al. Treating children for drug-resistant tuberculosis in Tajikistan with Group 5 medications. [Int J Tuberc Lung Dis.](https://www.ncbi.nlm.nih.gov/pubmed/?term=Treating+children+for+drug-resistant+tuberculosis+in+Tajikistan+with+Group+5+medications) 2016 Apr; 20(4): 474-8. | Retrospective cohort | All consecutively presenting bacteriologically confirmed and clinically diagnosed patients | Médecins Sans Frontières (MSF) Field Research Policy (<http://hdl.handle.net/10144/306501>); and data sharing repository ([data.sharing@msf.org](mailto:data.sharing@msf.org)) Amsterdam, Holland. Contact: Dr Sidney Wong, Medical Director, MSF Operational Centre Amsterdam, Holland ([Sidney.Wong@amsterdam.msf.org](mailto:Sidney.Wong@amsterdam.msf.org)) |
| Williams | United Kingdom | 28 | No |  | **Published** | Williams B, Ramroop S, Shah P, Anderson L, Das S, Riddell A, et al. [Multidrug-resistant tuberculosis in UK children: presentation, management and outcome.](http://www.ncbi.nlm.nih.gov/pubmed/23728407) Eur Respir J. 2013 Jun;41(6):1456-8. | Retrospective cohort | All consecutively presenting bacteriologically confirmed patients | Imperial College Research Ethics Committee, London, UK. Contact: http://www.imperial.ac.uk/research-ethics-committee// |
