# Supplementary material for: Treatment and outcomes in children with multidrug-resistant tuberculosis: A systematic review and individual patient data meta-analysis
Source: PLoS Med. 2018 Jul 11;15(7):e1002591. doi: 10.1371/journal.pmed.1002591 (PMC6040687; doi:10.1371/journal.pmed.1002591)
Supplement: S4 Table — (DOCX) [file pmed.1002591.s004.docx]

**S4 Table. Key clinical variables of children lost to follow up versus those with known treatment outcomes**

|  | **LTFU** | **N** | **Age** | | | | | | **Sex** | | | | | |  |  |  |  |  |  |
| --- | --- | --- | --- | --- | --- | --- | --- | --- | --- | --- | --- | --- | --- | --- | --- | --- | --- | --- | --- | --- |
|  |  |  | **<5** | **%** | **5 to <10** | **%** | **10 to 15** | **%** | **F** | **%** | **M** | **%** | **Missing** | **%** |  |  |  |  |  |  |
| All bacteriologically confirmed MDR-TB without XDR-TB (N=731) | No | **641** | 204 | 32% | 160 | 25% | 277 | 43% | 365 | 57% | 274 | 43% | 2 | 0% |  |  |  |  |  |  |
|  | Yes | **90** | 36 | 40% | 18 | 20% | 36 | 40% | 49 | 54% | 41 | 46% | 0 | 0% |  |  |  |  |  |  |
| All clinically confirmed MDR-TB (N=244) | No | **224** | 149 | 67% | 49 | 22% | 26 | 12% | 118 | 53% | 105 | 47% | 1 | 0% |  |  |  |  |  |  |
|  | Yes | **20** | 10 | 50% | 7 | 35% | 3 | 15% | 11 | 55% | 9 | 45% | 0 | 0% |  |  |  |  |  |  |
|  |  |  |  |  |  |  |  |  |  |  |  |  |  |  |  |  |  |  |  |  |
|  | **LTFU** | **N** | **HIV** | | | | | | **Severe Extra-Pulmonary Disease** | | | | | |  |  |  |  |  |  |
|  |  |  | **Yes** | **%** | **No** | **%** | **Missing** | **%** | **Yes** | **%** | **No** | **%** | **Missing** | **%** |  |  |  |  |  |  |
| All bacteriologically confirmed MDR-TB without XDR-TB (N=731) | No | **641** | 287 | 45% | 312 | 49% | 42 | 7% | 90 | 14% | 515 | 80% | 36 | 6% |  |  |  |  |  |  |
|  | Yes | **90** | 36 | 40% | 44 | 49% | 10 | 11% | 13 | 14% | 68 | 76% | 9 | 10% |  |  |  |  |  |  |
| All clinically confirmed MDR-TB (N=244) | No | **224** | 30 | 13% | 183 | 82% | 11 | 5% | 20 | 9% | 183 | 82% | 21 | 9% |  |  |  |  |  |  |
|  | Yes | **20** | 6 | 30% | 12 | 60% | 2 | 10% | 4 | 20% | 12 | 60% | 4 | 20% |  |  |  |  |  |  |
|  |  |  |  |  |  |  |  |  |  |  |  |  |  |  |  |  |  |  |  |  |
|  | **LTFU** | **N** | **Severe disease on chest radiograph** | | | | | | **Malnourished (low weight, edema or low weight for age)** | | | | | | **Previous Treatment** | | | | | |
|  |  |  | **Yes** | **%** | **No** | **%** | **Missing** | **%** | **Yes** | **%** | **No** | **%** | **Missing** | **%** | **Yes** | **%** | **No** | **%** | **Missing** | **%** |
| All bacteriologically confirmed MDR-TB without XDR-TB (N=731) | No | **640** | 360 | 56% | 155 | 24% | 125 | 20% | 244 | 38% | 337 | 53% | 60 | 9% | 250 | 39% | 320 | 50% | 71 | 11% |
|  | Yes | **91** | 47 | 52% | 13 | 14% | 31 | 34% | 32 | 36% | 44 | 49% | 14 | 16% | 33 | 37% | 35 | 39% | 31 | 34% |
| All clinically confirmed MDR-TB (N=244) | No | **224** | 62 | 28% | 119 | 53% | 43 | 19% | 49 | 22% | 167 | 75% | 8 | 4% | 29 | 13% | 178 | 79% | 17 | 8% |
|  | Yes | **20** | 5 | 25% | 7 | 35% | 8 | 40% | 7 | 35% | 8 | 40% | 5 | 25% | 2 | 10% | 12 | 60% | 6 | 30% |
