# Supplementary material for: Treatment and outcomes in children with multidrug-resistant tuberculosis: A systematic review and individual patient data meta-analysis
Source: PLoS Med. 2018 Jul 11;15(7):e1002591. doi: 10.1371/journal.pmed.1002591 (PMC6040687; doi:10.1371/journal.pmed.1002591)
Supplement: S5 Table — (DOCX) [file pmed.1002591.s005.docx]

S5 Table. Summary of association of use of individual drugs with treatment success in children treated for confirmed multidrug-resistant tuberculosis in South African study sites (MDR-TB; n=461) ^a,b,c,d^

| Drug Used | N (%) | aOR^e^ | 95% CI |
| --- | --- | --- | --- |
| Pyrazinamide | 449 (97) | 3.5^g^ | (0.3 -39.1) |
| **Second-line Injectable Agents^f^** | **430 (93)** | **8.09**^g^ | **(1.7-38.7)** |
| **Ethionamide/ prothionamide** | **448 (97)** | **11.7 ^g^** | **(1.5-89.1)** |
| **Cycloserine/ terizidone** | **212 (46)** | **3.0^g^** | **(1.4-6.1)** |
| Clofazimine | 7 (2) | 0.3 ^g^ | (0.02-3.4) |
| High-dose isoniazid | 130 (28) | 5.9 ^g^ | (0.9-20.6) |
| Para-aminosalicylic acid | 48 (10) | 0.8^g^ | (0.1-5.5) |
| Clarithromycin | 23 (5) | 0.5^g^ | (0.1-2.5) |
| Late-FQ |  | 0.3^g^ | (0.06-1.5) |

Treatment success was compared to Failure/Death by drug use

^a^The adjusted estimates for the clinically diagnosed children were not possible due to very low rates of failure

^b^Lost to follow-up was excluded from analysis

^c^All random effects (random intercept and random slope) models used maximum likelihood estimation with quadrature approximation, and were specified with an unstructured variance-covariance matrix parameterized through its Cholesky root unless otherwise stated.

^d^Too few children were treated with late generation fluoroquinolones, carbapenems and linezolid to be analysed. No children in these cohorts were treated with bedaquiline or delamanid.

^e^aOR, for use of drug, with non-use as reference category. Adjusted for age, sex, HIV infection, malnutrition, severity of disease on chest radiograph and severity of extrapulmonary disease

^f^Second-line injectable agents are amikacin, kanamycin and capreomycin

^g^Random-slope only model without random intercept, specified with standard variance components
