# Supplementary material for: Treatment and outcomes in children with multidrug-resistant tuberculosis: A systematic review and individual patient data meta-analysis
Source: PLoS Med. 2018 Jul 11;15(7):e1002591. doi: 10.1371/journal.pmed.1002591 (PMC6040687; doi:10.1371/journal.pmed.1002591)
Supplement: S6 Table — (DOCX) [file pmed.1002591.s006.docx]

S6 Table. Overview recent studies that may have been eligible for inclusion in this manuscript from October 2014 to present

| **Paper reference** | **Ages of subjects** | **Were subjects treated for multidrug-resistant tuberculosis (MDR-TB)?** | **Were treatment regimens reported?** | **Were treatment outcomes reported using standard WHO definitions?** | **Were there at least 3 eligible subjects (<15 years old, treated for MDR-TB with treatment regimens and outcomes reported)?** | **Would paper be eligible for inclusion in this manuscript?** | **Would inclusion of this paper in our analysis significantly affect the findings of our study?** |
| --- | --- | --- | --- | --- | --- | --- | --- |
| Chiang et al. Baseline Predictors of Treatment Outcomes in Children With Multidrug-Resistant Tuberculosis: A Retrospective Cohort Study. [Clin Infect Dis.](https://www.ncbi.nlm.nih.gov/pubmed/27458026) 2016 Oct 15;63(8):1063-71. | <= 15 years old | Yes | Yes | Yes | Yes | Yes | It is unlikely that inclusion of the data in this paper would change our findings. The paper included 115 subjects with confirmed MDR-TB and 117 with probable MDR-TB. However, major findings did not differ. Treatment success rates were similar to our study (70%). The study data was drawn from years 2005-2009, like the majority of our data and therefore no new medications or diagnostic methods were used in this paper that were not used in our manuscript. This paper did not find an association between the baseline treatment regimen and treatment outcomes. This paper’s predictors for poor outcome were severe disease and malnutrition, which were similar to our findings. They did not have enough HIV cases to analyze possible affects from this. |
| Galli L et al. Pediatric Tuberculosis in Italian Children: Epidemiological and Clinical Data from the Italian Register of Pediatric Tuberculosis. [Int J Mol Sci.](https://www.ncbi.nlm.nih.gov/pubmed/27322255) 2016 Jun 17;17(6). | 0-18 years old | Yes | Yes | Yes | Possible, there were 10 children <18 years old with MDR –TB but only 6 were treated with at least 4 second-line medications. | Yes | Data was collected from 2010-2012; 25% of our data comes from 2010 and later, so this data does not represent a novel time frame for this manuscript. None of the new medications were used, although a handful of patients did receive linezolid or moxifloxacin. However, since at most only 10 children from this paper could have been included in our manuscript, it is unlikely that inclusion of this data would have changed our findings. |
| Malik R et al. Childhood abdominal tuberculosis: Disease patterns, diagnosis, and drug resistance. [Indian J Gastroenterol.](https://www.ncbi.nlm.nih.gov/pubmed/26678593) 2015 Nov;34(6):418-25. | <16 years old | Yes | Unclear | Unclear | Possible, although it is unclear if there were 3 children <15 years old and if treatment regimen and outcome data were adequately reported. | Potentially | It is unlikely that data from this paper would have changed our findings. At most, only 3 children could have been included and the data were from years 2000 -2012, which is a time frame well covered in our manuscript and would not have included any of the new drugs. |
| Meressa D et al. Achieving high treatment success for multidrug-resistant TB in Africa: initiation and scale-up of MDR-TB care in Ethiopia--an observational cohort study. [Thorax.](https://www.ncbi.nlm.nih.gov/pubmed/26506854) 2015 Dec;70(12):1181-8. | All ages included, 5% of the 612 total patients (31 patients) were<18 years old | Yes | Yes | Yes | It is likely there were at least 3 children < 15 years old | Likely | It is unlikely that inclusion of the data in this paper would change our findings. There would have been at most 31 children that could have been included. However, major findings did not differ from our manuscript. Treatment success rates for patients < 18 years old were similar to our study (80%). Total rates of HIV infection were 21%. Only 6 subjects (and it is unknown if they were pediatric patients or not) could have used “newer” medications including moxifloxacin, linezolid or bedaquiline, so this paper would not have added to our analysis of treatment outcomes. This paper’s predictors for poor outcome were malnutrition, HIV infection and confirmed MDR-TB (and cor pulmonale) were similar to our findings. |
| Achar J et al. Off-Label Use of Bedaquiline in Children and Adolescents with Multidrug-Resistant Tuberculosis. [Emerg Infect Dis.](https://www.ncbi.nlm.nih.gov/pubmed/28758889) 2017 Oct;23(10). | Median age 16 years old, range 10-17 years old | Yes | Yes | Yes | Yes, 9 patients had confirmed MDR and 10 were clinically diagnosed. | Yes | This paper does analyze the treatment outcomes of patients treated with many of the new drugs, including bedaquiline, a drug that was not used in our cohort. However, with only 17 patients with bacteriologically confirmed disease, it is unlikely that this would have made a statistically significant impact on our findings. |
| Elmi OS. Treatment Outcomes of Patients with Multidrug-Resistant Tuberculosis (MDR- TB) Compared with Non-MDR-TB Infections in Peninsular Malaysia. [Malays J Med Sci.](https://www.ncbi.nlm.nih.gov/pubmed/27660541) 2016 Jul;23(4):17-25. | 23 patients with MDR-TB. Median age 40.4 years, IQR 14.75 years. Pediatric population not well defined. | Yes | It is not entirely clear but it appears no data on treatment regimens were collected. | Yes | Unlikely, it appears that data on treatment regimens were not collected and given the median age and IQR, it is unlikely that enough children were present to include this study in our study. | Unlikely | It is unlikely that there would have been enough children in this study to affect our findings. |
