# Supplementary material for: Treatment and outcomes in children with multidrug-resistant tuberculosis: A systematic review and individual patient data meta-analysis
Source: PLoS Med. 2018 Jul 11;15(7):e1002591. doi: 10.1371/journal.pmed.1002591 (PMC6040687; doi:10.1371/journal.pmed.1002591)
Supplement: S2 Text — (DOCX) [file pmed.1002591.s008.docx]

**S2 Text: Study Protocol**

**Evidence synthesis to inform the paediatric component of revised WHO guidelines on the management of multidrug-resistant tuberculosis**

September 16, 2014

**Coordinating Center**

Desmond Tutu TB Centre

Department of Paediatrics and Child Health

Faculty of Medicine and Health Sciences

Stellenbosch University

Cape Town, South Africa

**Key Personnel**

- Anneke Hesseling (Stellenbosch University, clinician)
- Anthony Garcia-Prats (Stellenbosch University, clinician)
- H. Simon Schaaf (Stellenbosch University, clinician)
- Nathan Ford (methodology expert, statistician, WHO HIV department)
- Elizabeth Harausz (ID fellow, Case Western University)
- Jennifer Furin (Case Western Reserve University and Sentinel project, clinician)
- James Seddon (Imperial College, London, clinician)
- Tamara Kredo (South African MRC, Cochrane Centre, systematic review methodology expert)
- Helen McIlleron (University of Cape Town, clinical pharmacologist)
- Additional expertise may be co-opted as necessary

Table of Contents

[List of Abbreviations 4](#_Toc398595318)

[Background and scientific rationale 5](#_Toc398595319)

[Systemic Review and Individual Patient Data Meta-Analysis 5](#_Toc398595320)

[Objectives 5](#_Toc398595321)

[Methods 6](#_Toc398595322)

[*Study Designs Eligible for Inclusion* 6](#_Toc398595323)

[*Description of Participants* 6](#_Toc398595324)

[*Case definitions* 7](#_Toc398595325)

[*Interventions* 7](#_Toc398595326)

[*Specific Primary Objectives* 7](#_Toc398595327)

[*Outcome Measures* 8](#_Toc398595328)

[*Primary outcome* 8](#_Toc398595329)

[*Secondary outcomes* 8](#_Toc398595330)

[*Search Strategy* 8](#_Toc398595331)

[*Data Collection* 9](#_Toc398595332)

[*Data extraction and management* 9](#_Toc398595333)

[*Information required* 10](#_Toc398595334)

[*Data Analysis* 10](#_Toc398595335)

[*Assessment of Heterogeneity* 10](#_Toc398595336)

[*Sub-Group Analyses* 10](#_Toc398595337)

[*Assessing Quality of Included Studies/Risk of Bias* 11](#_Toc398595338)

[*Assessment of Quality of Evidence Across Studies* 11](#_Toc398595339)

[Ethics 11](#_Toc398595340)

[*Risks to Subjects Included in the Study* 11](#_Toc398595341)

[*Benefit to Subjects Included in Study* 12](#_Toc398595342)

[Scope of Work 12](#_Toc398595343)

[References 13](#_Toc398595344)

[Appendix A: Study Objectives within PICO format 15](#_Toc398595345)

[Appendix B: Definitions for Study Classifications 16](#_Toc398595346)

[Appendix C: General Information from Each Reporting Site/Centre 18](#_Toc398595347)

[Appendix D: Information for Each Patient 19](#_Toc398595348)

# List of Abbreviations

| AFB | Acid-fast bacilli |
| --- | --- |
| ART | Antiretroviral treatment |
| DOT | Directly observed therapy |
| DST | Drug susceptibility testing |
| DR-TB | Drug-resistant tuberculosis |
| EML | Essential Medicines List |
| HIV | Human immunodeficiency virus |
| INH | Isoniazid |
| IPD | Individual patient data |
| MDR-TB | Multidrug-resistant tuberculosis |
| MTB | Mycobacterium tuberculosis |
| MUAC | Mid-upper arm circumference |
| PK | Pharmacokinetics |
| PICO | Population and Patients/ Intervention/Comparator/Outcome |
| RMR | Rifampicin-monoresistant |
| TB | Tuberculosis |
| WHO | World Health Organization |
| XDR-TB | Extensively drug-resistant tuberculosis |

# Background and Scientific Rationale

Multidrug-resistant tuberculosis (MDR-TB) in children is under recognized, under diagnosed and under treated. Despite the fact that approximately 32,000 children develop MDR-TB each year [9]and that historical studies have shown mortality rates from TB of 40%, 16% and 5%, respectively, for infants, toddlers and young children [10] , very little is known about the optimal treatment for children infected with MDR-TB. Treatment for MDR-TB is difficult, requiring use of toxic medications for at least 18 months and with only 80% of children achieving successful outcomes [1]. Therefore, more data is needed to help improve MDR-TB treatment regimens. A systemic review in 2012 sought to better quantify treatment outcomes in children [1], however, many questions remain on how to optimize successful treatment outcomes and minimize adverse events. This systemic review and meta-analysis will seek to inform updated guidelines on the management of children with MDR-TB, by describing key outcomes among children treated for MDR-TB and addressing questions specifically relevant to the paediatric population with MDR-TB. To achieve this, we will complete a systematic review and individual patient data (IPD) meta-analysis of MDR-TB treatment regimens and outcomes in children.

# Systemic Review and Individual Patient Data Meta-Analysis

# Objectives

The objectives of this review are to evaluate the safety and efficacy of various MDR-TB treatment regimens in children diagnosed with MDR-TB. This will be addressed through the formation of several specific questions regarding paediatric MDR-TB management and treatment. Anticipated limitations in analyzing and interpreting paediatric data include the lack of standardization of MDR-TB therapy. Treatment may be individualized according to several factors such as disease severity (e.g. time of diagnosis, *Mycobacterium tuberculosis* (MTB) burden, extent of disease) and drug susceptibility testing (DST) results. The collection and analysis of the available paediatric data into an individual patient database will help unite individual patient outcomes into evidence for treatment guidelines. Additional considerations for interpretation of paediatric MDR-TB data include the lack of bacteriological confirmation in a substantial proportion of cases and the definition of successful treatment outcome.

# Methods

Methods will follow the previously completed adult review [1], as far as possible, with consideration of paediatric specific aspects in the analysis (e.g. case definitions, disease severity and treatment outcomes).

There is limited data on the programmatic management and outcomes of children treated for MDR-TB. A systematic review of children treated for MDR-TB published in 2012 included eight studies with 315 total patients [1]. This review did not include IPD meta-analysis. Additional studies have since been published. An updated review will be important for characterizing current management strategies and outcomes to inform updated MDR-TB treatment guidelines in children. We propose to conduct a systematic review of the available literature of children treated for MDR-TB.

An IPD meta-analysis for (predominately) adult MDR-TB treatment outcomes was published in 2012 [2]. An IPD meta-analysis based on pediatric data can be used to estimate associations of different treatment regimens with treatment outcome and other clinically relevant outcomes (e.g. toxicity) in children, with consideration of clinically relevant covariates of interest. We propose to conduct an IPD meta-analysis from the studies included in the overall updated systematic review. This will provide an updated evidence synthesis that is harmonized with the previously completed adult review, and will allow us to address specific questions relevant to paediatric MDR-TB treatment as specified below.

## *Study Designs Eligible for Inclusion*

Studies will be eligible if they included more than five children (aged ≤16 years for the systemic review, aged ≤ 14 years for the IPD meta-analysis) within a defined treatment cohort. Both controlled and non-controlled retrospective and prospective studies will be included. In anticipation of a paucity of data, case series of five or more children will also be included. Unpublished data will also be included, as long as its collection was approved by the ethics board of its originating institution.

## *Description of Participants*

For the IPD meta-analysis, only children 0-14 years old will be included. This age group was chosen because historical data shows that children up to this age range have drastically different outcomes, with higher mortality, than adults [10]. For the systematic review, the population examined will be children between 0-16 years . This age group will be used for the systemic review as frequently studies include children up to 16 years old, and given the paucity of paediatric MDR-TB data, we want to avoid the exclusion of otherwise appropriate studies. It is expected that, due to anticipated low numbers of children, including studies that include children up to 16 years old will not significantly affect the review. The children must be treated for clinically diagnosed or bacteriologically confirmed pulmonary or extrapulmonary MDR-TB, using standard WHO TB case definitions [4, 5].

### *Case definitions*

Please see Appendix B for complete study definitions regarding diagnosis of TB. Children meeting the following criteria will be considered as having MDR-TB:

- Bacteriologically confirmed: based on confirmed MDR DST results from a MTB isolate or genotypic result *or*
- Clinically diagnosed MDR-TB: based on the provision of second-line TB drugs for at least 2 months together with either clinical evidence of tuberculosis or a known contact with MDR-TB disease.

Clinically diagnosed TB will be further categorized, where possible, using standard research diagnostic criteria for paediatric TB (see Appendix B).

Children with an isolated GeneXpert MTB/RIF result indicating rifampicin resistance, but in the absence of confirmed additional isoniazid resistance, will be analyzed as a subgroup.

## *Interventions*

### *Specific Primary Objectives*

1. To evaluate the efficacy and safety of MDR-TB treatment in children between 0-16 years of age with clinically or bacteriologically confirmed pulmonary or extrapulmonary MDR- TB, using standard WHO TB case definitions. (see Appendix B)

We will describe the regimens and related treatment outcomes in children with MDR-TB, and will address specific questions detailed below:

*Question 1*: Among children treated for MDR-TB, do regimens containing ≥6 drugs, given at least during the intensive phase of treatment, result in a similar proportion of successful treatment outcomes compared to regimens containing <6 drugs?

*Question 2:* Among children treated for MDR-TB, do regimens using <4 months of a second-line injectable drug (amikacin, kanamycin, or capreomycin) result in a similar proportion of successful treatment outcomes compared to regimens using ≥4 months of a second-line injectable drug?

*Question 3:* Among children treated for MDR-TB, do total treatment durations of <18 months, result in a similar proportion of successful treatment outcomes compared to total treatment durations of ≥18 month regimens?

*Question 4:* Among children treated for MDR-TB, does the inclusion of a later generation fluoroquinolone (moxifloxacin or levofloxacin) in the treatment regimen, result in a similar proportion of successful treatment outcomes compared to treatment with an earlier generation fluoroquinolone (ofloxacin or ciprofloxacin)?

*Question 5:* Among children treated for MDR-TB, does the inclusion of isoniazid (high-dose, i.e. 20 mg/kg/day) in the treatment regimen, result in a similar proportion of successful treatment outcomes compared to treatment without isoniazid (high-dose)?

*Question 6:* Among children treated for MDR-TB, does the inclusion of ethionamide/prothionamide in the treatment regimen, result in a similar proportion of successful treatment outcomes compared to treatment without ethionamide/prothionamide?

See Appendix A for study objectives displayed in PICO format.

## *Outcome Measures*

### *Primary outcome*

1. TB treatment outcome: Successful treatment outcomes (cure or treatment completed) and unsuccessful outcome (treatment failure, death, etc) as defined by WHO [4,5] (see detailed definitions in Appendix B)

### *Secondary outcomes*

1. Death [4,5]
2. Lost to follow-up/not evaluated [4,5]
3. 2-month sputum culture conversion among those with bacteriologically confirmed TB
4. Clinically significant adverse events (e.g. optic neuritis/peripheral neuropathy; thyroid dysfunction; hepatotoxicity and arthralgia/arthritis)
5. Drug cost would be calculated but formal costing and health economics evaluation is beyond the scope of this review.

## *Search Strategy*

We will conduct a comprehensive search for all relevant evidence, regardless of language or publication status. We will include unpublished data if it has been approved by the ethics committee at it originating institution. We will search the following databases:

PubMed, LILACS, Embase, *The Cochrane Library*, PsychINFO, and BioMedCentral databases up to 30 September 2014, with a search strategy, using a combination of the search terms “tuberculosis”, “multidrug resistance”, “MDR-TB”, “multidrug-resistant”, and “children”, both as exploded MESH headings and free-text terms, and without language restriction.

We will work with a search expert, and consider the following search terms: TUBERCULOSIS MULTIDRUG-RESISTANT; multidrug-resistant tuberculosis; MDR tuberculosis; MDR-TB; multi-drug resistant tuberculosis; drug-resistant tuberculosis; multiple drug resistant tuberculosis

We will search all electronically available conference abstracts from the International Union Against Tuberculosis and Lung Disease (2004–2014).

We will review the bibliographies of all retrieved articles and also contact experts in the field, to identify any additional studies.

We will search for ongoing studies or clinical trials on the WHO International Clinical Trials Registry Platform (ICTRP).

## *Data Collection*

Two authors will independently select potentially relevant studies by scanning the titles, abstracts, and descriptor terms of the references found by the search. They will apply the inclusion criteria as defined above. Irrelevant reports will be discarded, and the full article or abstract obtained for all potentially relevant or uncertain reports. The two authors will independently apply the inclusion criteria. Studies will be reviewed for relevance, based on study design, types of participants, exposures and outcomes measures. A third party will adjudicate any disagreements that could not have been resolved by discussion. Where there is missing data, authors of relevant studies will be contacted for clarification and additional information.

### *Data extraction and management*

After initial search and article screening, two reviewers will independently extract information from each selected study onto standardized data extraction form. Extracted information will include (See Appendix C and D for complete list):

- Study details: citation, start and end dates, location, study design.
- Site details: type of health care centre, TB culture and DST methods, ability to assess for adverse events.
- Baseline patient characteristics: age, gender, date of TB diagnosis, nutritional status, HIV status
- Diagnostics: details regarding diagnosis of MDR-TB and site and extent of disease
- Intervention details: duration and medications used in MDR-TB treatment
- Outcome details: treatment outcomes, length of time to culture and smear conversion, length of follow-up, adverse events.

After identification of eligible primary studies/cohorts, individual patient data will be requested from the authors of the studies selected for inclusion. Studies/cohorts included in the IPD analysis will meet the following inclusion criteria:

(i) Investigators agree to collaborate and can provide data in electronic format within 3 months.

(ii) Information is complete for at least 75% of patients with the essential information (listed below).

(iii) At least one of the 4 key treatment outcomes (i.e. cure, treatment completion, treatment failure, died, lost to follow-up) were measured and reported.

Defaulted treatment will be classified as not taking treatment for 2 months or more.

### *Information required*

If not available in the publication, the information will be requested from individual authors. Information requested will pertain either to the study site or individual patient (see Appendix C and D for information that will be requested from authors).

## *Data Analysis*

We will use STATA and Excel for statistical analysis and GRADEpro software ([GRADEpro 2008](file:///C:\Users\tkredo\Desktop\GRADEpro%202008)) to produce GRADE Summary of Findings tables and GRADE Evidence Profiles. We will summarize dichotomous outcomes for effect in terms of risk ratio (RR) with their 95% confidence intervals. We will calculate summary statistics using meta-analytic methods and present findings in GRADE Summary of Findings tables and GRADE Evidence Profiles for all outcomes of interest.

### *Assessment of Heterogeneity*

For outcomes that undergo meta-analysis, we will examine heterogeneity by using the χ^2^ statistic with a significance level of 0.10, and the I^2^ statistic. We will interpret an I^2^ estimate greater than 50% as indicating moderate or high levels of heterogeneity and will investigate its causes ([8](file:///C:\Users\tkredo\Desktop\Deeks%202008)).

### *Sub-Group Analyses*

The influence of these pre-specified covariates on treatment outcome will be examined in the secondary analyses:

1. Disease Severity:
   1. Presence of cavities on chest x-ray (and CXR findings where available)
   2. Pulmonary vs extrapulmonary tuberculosis (and type of extrapulmonary TB)
   3. Disseminated tuberculosis (TB meningitis, miliary disease and other disseminated disease).
   4. Proposed consensus definitions for severe vs. non-severe TB in children
2. Age (strata: 0-4 years vs 5-14 years, as per WHO reporting categories)
3. Gender
4. HIV co-infection
5. Culture status (positive growth or no growth)
6. Smear status (positive for acid fast bacilli (AFB)on smear or no AFB seen)

3. GeneXpert status

4. Second-line anti-TB drug resistance (specifically to amikacin , kanamycin, capreomycin and fluoroquinolones, i.e. XDR-TB)

5. Rifampicin mono-resistance (confirmed rifampicin mono-resistance will be excluded in the IPD)

6. Identification of isoniazid (INH) resistance gene mutation

7. Nutritional status

8. Delivery of standardized treatment regimens versus treatment regimens individualized by DST (either DST of the patient’s isolate or of their TB contact), if available.

9. DOT (directly observed therapy) use

10. Hospital-based vs. ambulatory care

### *Assessing Quality of Included Studies/Risk of Bias*

Two review authors will independently assess the risk of bias within the included studies against key criteria described below. We will resolve disagreements by consensus, or involve an arbitrator when necessary.

*Controlled Cohort Studies*

We will use the Newcastle-Ottawa Scale ([Newcastle-Ottawa Scale](file:///C:\Users\tkredo\Desktop\Newcastle-Ottawa%20Scale)) to assess the quality and risk of bias in non-randomized studies. Specifically, the scale uses a star system to judge three general areas: selection of study groups, comparability of groups, and ascertainment of outcomes (in the case of cohort studies). As a result, this instrument can assess the quality of non-randomized studies so that they can be used in a meta-analysis or systematic review.

### *Assessment of Quality of Evidence Across Studies*

We will assess the quality of evidence across a body of evidence (i.e., multiple studies with similar interventions and outcomes) with the GRADE approach ([6](file:///C:\Users\tkredo\Desktop\Guyatt%202011)), defining the quality of evidence for each outcome as “the extent to which one can be confident that an estimate of effect or association is close to the quantity of specific interest” ([7](file:///C:\Users\tkredo\Desktop\Higgins%202008)). The quality rating across studies has four levels: high, moderate, low or very low. Randomized controlled trials are initially categorized as providing high quality evidence, but the quality can be downgraded; similarly, other types of controlled trials and observational studies are initially categorized as providing low quality evidence, but the quality can be upgraded. Factors that decrease the quality of evidence include limitations in design, indirectness of evidence, unexplained heterogeneity or inconsistency of results, imprecision of results or high probability of publication bias. Factors that can increase the quality level of a body of evidence include a large magnitude of effect, if all plausible confounding would lead to an underestimation of effect and if there is a dose-response gradient.

# Ethics

No additional data will be collected for this purpose of this study. This evidence synthesis will combine already collected and disseminated data. Only data that has been approved by the ethics committee at the original institution will be considered for inclusion in the study. Data sharing agreements will be completed, along with central ethics approval at one central research ethics committee (Stellenbosch University). All collaborating investigators will be invited to share publication credit and will be acknowledged accordingly.

## *Risks to Subjects Included in the Study*

The risk to subjects included in this study is the loss of confidentiality of personal health information. However, subject confidentiality will be maintained to the best of our ability. Subject names or any other personal identifiers will not be used or entered into the database. Anonymous patient identifier numbers will be used instead. We will request that all authors remove any personal identifiers from their data before sending their data to us. Study-related information will be kept confidential.

## *Benefit to Subjects Included in Study*

There is no direct benefit for subjects included in this study. However, knowledge gained from this study may advance understanding of the optimal methods to treat childhood MDR-TB. This knowledge may lead to improved care that will benefit future children infected by MDR-TB.

# Scope of Work

We therefore propose to complete these 2 interrelated systematic reviews.

1. Systematic review and meta-analysis of MDR-TB treatment regimens and outcomes in children

2. Individual patient data meta-analysis of MDR-TB treatment regimens and outcomes in children

# References

1. Ettehad D, Schaaf HS, Seddon JA, Cooke GS, Ford N. Treatment outcomes for children with multidrug resistant tuberculosis: a systemic review and meta-analysis. Lancet Infec Dis 2012; 12: 449-56.

2. Ahuja SD, Ashkin D, Avendano M, Banerjee R, Bauer M, Bayona JN, Becerra MC, Benedetti A, Burgos M, Centis R, Chan ED, Chiang CY, Cox H, D'Ambrosio L, DeRiemer K, Dung NH, Enarson D, Falzon D, Flanagan K, Flood J, Garcia-Garcia ML, Gandhi N, Granich RM, Hollm-Delgado MG, Holtz TH, Iseman MD, Jarlsberg LG, Keshavjee S, Kim HR, Koh WJ, Lancaster J, Lange C, de Lange WC, Leimane V, Leung CC, Li J, Menzies D, Migliori GB, Mishustin SP, Mitnick CD, Narita M, O'Riordan P, Pai M, Palmero D, Park SK, Pasvol G, Peña J, Pérez-Guzmán C, Quelapio MI, Ponce-de-Leon A, Riekstina V, Robert J, Royce S, Schaaf HS, Seung KJ, Shah L, Shim TS, Shin SS, Shiraishi Y, Sifuentes-Osornio J, Sotgiu G, Strand MJ, Tabarsi P, Tupasi TE, van Altena R, Van der Walt M, Van der Werf TS, Vargas MH, Viiklepp P, Westenhouse J, Yew WW, Yim JJ; Collaborative Group for Meta-Analysis of Individual Patient Data in MDR-TB. [Multidrug resistant pulmonary tuberculosis treatment regimens and patient outcomes: an individual patient data meta-analysis of 9,153 patients.](http://www.ncbi.nlm.nih.gov/pubmed/22952439) PLoS Med. 2012;9(8):e1001300. Epub 2012 Aug 28. PMID: 22952439

3. Seddon JA, Perez-Velez CM, Schaaf HS, Furin JJ, Marais BJ, Tebruegge M, Detjen A, Hesseling AC, Shah S, Adams LV, Starke JR, Swaminathan S, Becerra MC; on Behalf of the Sentinel Project on Pediatric Drug-Resistant Tuberculosis. [Consensus Statement on Research Definitions for Drug-Resistant Tuberculosis in Children.](http://www.ncbi.nlm.nih.gov/pubmed/23717785) J Pediatric Infect Dis Soc. 2013 Jun;2(2):100-109. Epub 2013 Apr 10. PMID: 237177854.

4. Guidance for national tuberculosis programmes on the management of tuberculosis in children: second edition. 2014. [internet]. World Health Organization (WHO), 2014. Geneva. Available from: <http://www.who.int/tb/publications/childtb_guidelines/en/>

5. Definitions and reporting framework for tuberculosis : 2013 revision. World Health Organization (WHO), 2013. Geneva.

6.Guyatt GH, Oxman AD, Schunemann HJ, Tugwell P, Knottnerus A. GRADE guidelines: a new series of articles in the Journal of Clinical Epidemiology. Journal of Clinical Epidemiology 2011;64(4):380-2. [PubMed: 21185693]

7. Higgins JPT, Green S (editors). Cochrane Handbook for Systematic Reviews of Interventions Version 5.1.0 [updated March 2011]. The Cochrane Collaboration, 2011. Available from: http://www.cochrane-handbook.org, (Accessed 20 February 2012).

1. Deeks JJ, Higgins JPT, Altman DG. Chapter 9: Analysing data and undertaking meta-analyses. In: Higgins JPT, Green S, editor(s). Cochrane Handbook for Systematic Reviews of Interventions. Chichester: John Wiley & Sons, 2008:276-82.
2. Jenkins HE, Tolman AW, Yuen CM, Parr JB, Keshavjee S, Pérez-Vélez CM, Pagano M, Becerra MC, Cohen T. Incidence of multidrug-resistant tuberculosis disease in children: systematic review and global estimates. Lancet. 2014 May 3; 383(9928):1572-9.
3. Wallgren A. Primary tuberculous infections in young adult life and in childhood. Am J Dis Child 1941; 61: 577-589

# Appendix A: Study Objectives within PICO format

**Table 1. PICO components**

| **Population/**  **Patients** | **Intervention** | **Comparator** | **Outcome/s** |
| --- | --- | --- | --- |
| Primary population:   - Children, age 0-14 (or to 16 years, see *Methods*) years, treated for clinically diagnosed or bacteriologically confirmed pulmonary or extrapulmonary MDR-TB   Subgroups/other covariates of interest (as specified above) | 1. Treated with ≥6 drugs during at least the intensive phase of treatment | 1. Treated with <6 drugs during at least the intensive phase of treatment | Primary outcome:  Treatment success (cure or treatment completed) and treatment failure.  Secondary outcomes:   1. Death 2. Lost to follow-up/ not evaluated 3. 2 month sputum conversion among those with bacteriologically confirmed TB 4. Clinically significant adverse events 5. Drug cost   Note: in addition to standard WHO treatment outcomes, international research consensus for paediatric MDR-TB treatment outcomes will also be described. |
|  | 1. Treated with second-line injectable TB drugs for <4 months | 1. Treated with second-line injectable TB drugs for ≥4 months |  |
|  | 1. Treated for a total duration of <18 months | 1. Treated for a total duration of ≥18 months |  |
|  | 1. Use of later generation fluoroquinolone (moxifloxacin or levofloxacin) | 1. Use of earlier generation fluoroquinolone (ofloxacin or ciprofloxacin) |  |
|  | 1. Inclusion of high dose isoniazid in treatment regimen | 1. Treatment regimens without high dose isoniazid |  |
|  | 1. Inclusion of ethionamide/ prothionamide in treatment regimen | 1. Treatment regimens without ethionamide/ prothionamide |  |

# Appendix B: Definitions for Study Classifications

Certainty of Diagnosis of TB Disease (as per WHO guidelines)[5]

- Confirmed: A bacteriologically confirmed TB case is one from whom a biological specimen is positive by smear microscopy, culture or WHO approved rapid diagnostics (such as Xpert MTB/RIF or LPA).
- Clinically diagnosed case: a case who does not fulfill the criteria for bacteriological confirmation but who has been diagnosed with TB disease by a clinician or other medical practitioner who has decided to give the patient a full course of MDR-TB treatment.

In addition to these WHO diagnostic categories, the certainty of diagnosis will also be reported using International Consensus Statement definitions for paediatric TB [3].

- Confirmed TB disease: At least 1 of the signs and symptoms suggestive of TB disease and microbiological confirmation of MTB
- Probable TB disease: At least one of the signs and symptoms suggestive of TB disease and the chest X-ray is consistent with intrathoracic TB disease and presence of one of the following:
  - Positive clinical response to TB treatment
  - Documented exposure to a source case with TB disease
  - Immunological evidence of TB disease
- Possible TB disease: At least one of the signs and symptoms suggestive of TB disease and either:
  - Clinical response to TB treatment, documented exposure to a source case with TB disease or immunological evidence of TB infection
  - Chest X-ray consistent with intrathoracic TB disease.

Drug Resistance Categories (as per WHO guidelines)[5]

- Monoresistance: Resistance to one first-line anti-TB drug only
- Polydrug resistance: resistance to more than one first-line anti-TB drug (other than both rifampicin and isoniazid)
- Multidrug resistance: Resistance to at least both rifampicin and isoniazid
- Extensive drug resistance: resistance to any fluoroquinolone and to at least one of the three second-line injectable drugs (capreomycin, kanamycin and amikacin), in addition to multidrug resistance.
- Rifampicin resistance: resistance to rifampicin detected using phenotypic or genotypic methods, with or without resistance to other anti-TB drugs. It includes any resistance to rifampicin, whether monoresistance, multidrug resistance, polydrug resistance or extensive drug resistance

Treatment Outcomes (As per WHO Guidelines) [5]

- Cured: Treatment completed as recommended by the national policy without evidence of failure and three or more consecutive cultures taken at least 30 days apart (standard paediatric definitions; WHO adult recommendations are 5 or more consecutive cultures) are negative after the intensive phase
- Treatment completed: treatment completed as recommended by the national policy without evidence of failure but no record that three or more consecutive cultures taken at least 30 days apart are negative after the intensive phase
- Treatment failed: treatment terminated or need for permanent regimen change of at least two anti-TB drugs because of:
  - Lack of conversion by the end of the intensive phase *or*
  - Bacteriological reversion in the continuation phase after conversion to negative *or*
  - Evidence of additional acquired resistance to fluoroquinolones or second-line injectable drugs *or*
  - Adverse drug reactions resulting in discontinuation of at least 2 antituberculosis drugs.
- Died: A patient who dies for any reason during the course of treatment
- Lost to follow-up: a patient whose treatment was interrupted for 2 consecutive months or more
- Not evaluated: A patient for whom no treatment outcome is assigned. This includes cases “transferred out” to another treatment unit and whose treatment outcome is unknown
- Treatment success: the sum of *cured* and *treatment completed*

In addition to standard WHO treatment outcomes, the following treatment outcomes as per International TB Consensus Statement definitions for Paediatric TB [3] may also be considered in analysis:

- Probable cure: completion of prescribed treatment with attainment of clinical and radiological improvement
- Default: treatment interruption for 2 months or more
- Primary default: never started on DR-TB treatment
- Primary death: death before starting DR-TB treatment

# Appendix C: General Information from Each Reporting Site/Centre

Type of centre (specialized, tertiary care hospital seeing only referred patients, or programme wide and population based)

Patient selection

- - How are MDR patients identified (i.e. all new cases get DST, or only failures, or failures of retreatment, etc).
  - Were all MDR patients identified or treated included in the series/report?

Lab methods

- - What is the method of TB culture and DST? The latter includes critical concentrations if phenotypic testing. Were molecular methods used for TB diagnosis and DST? If so, specific which (e.g. Xpert MTB/RIF, line probe assay etc)

Treatment

- - A detailed and complete definition of treatment outcomes used in the study, at each centre.
  - Is the treatment regimen individualized on the basis of DST, or standardized.
  - How long are children with MDR-TB typically followed? (for IPD, the last date seen or number of months after treatment completion will be recorded)

Assessment of adverse events

- - Which safety monitoring tests were done as a routine (without obvious clinical symptoms)
  - Which tests were done only when there was clinical indication of a toxicity
  - What would criteria for discontinuation of TB treatment typically be in relation to adverse events?

# Appendix D: Information for Each Patient

*Baseline patient characteristics*

Essential data include all of the following:

- Age at TB diagnosis
- Gender
- HIV sero-status (*missing data may be acceptable if the sero-prevalence of HIV was known to be very low in the population of TB patients treated at the same time in the same centre),*
- Weight at time of start of treatment

Desirable

- Certainty of diagnosis of TB disease per Consensus Statement: confirmed TB disease, probable TB disease, possible TB disease (see *Definitions for Study Classification*)
- Previous TB treatment: drugs used, date(s) outcomes
- Comorbidities
- Use of ART, and if so, what ART regimen (and timing of initiation)
- Nutritional status: height, weight, clinically diagnosed with malnutrition, mid-upper arm circumference (MUAC), presence of nutritional oedema

*Disease site and severity factors*

Essential: all of the following:

- Site of disease (especially pulmonary, extrapulmonary, both)
- AFB smear results
- Culture results

(molecular tests e.g. Xpert MTB/Rif would also be included).

Desirable

- Chest X-ray findings (e.g. presence of cavities and other features)

*Drug Susceptibility Testing (DST)*

Essential

- DST results for isoniazid and rifampicin

Desirable

- DST results for other first line drugs (ethambutol, pyrazinamide, streptomycin)
- Second-line DST results (particularly to second line injectables and fluoroquinolones)
- GeneXpert results
- Characterization of the INH genetic mutation present

**Treatment factors**:

Essential: all of the following:

- Actual drug regimen - specific drugs used, and their duration in intensive and continuation phases. If INH used, specify if high dose or regular dose used.
- Surgical resection (if any)
- Regimen modified (or not) in response to first or second line DST results.
- Use of DOT

Desirable

- Mode of supervision of therapy
- Dose of each drug (and body weight of patients – to calculate dose in mg/kg).
- Hospital admission and duration

*Treatment outcomes*

Essential: Classified as at least one of the following:

- Cure
- Treatment completion
- Treatment failure
- Died
- Lost to follow-up
- Not evaluated

Desirable:

- Probable cure, default, primary default, primary death(as per consensus statement definitions (See *Definitions for Study Classification*, Consensus Statement definitions)
- Relapse
- Clinically significant adverse events
  - hearing loss (diagnosed via audiology or clinically)
  - neurotoxicity (including peripheral neuropathy and optic neuropathy)
  - thyroid dysfunction (diagnosed as per treating providers decision to supplement thyroid hormone based on clinical and/or lab evaluation)
  - liver toxicity (diagnosed either by abnormal liver tests or clinical diagnosis)
  - cartilage toxicity (per clinical diagnosis)
  - nephrotoxicity
- Microbiologic conversion (time to culture or smear conversion, if culture is not available, or % with sputum smear and culture conversion at fixed intervals)
